# Geminin prevents DNA damage in vagal neural crest cells to ensure normal enteric neurogenesis

**DOI:** 10.1186/s12915-016-0314-x

**Published:** 2016-10-24

**Authors:** Chrysoula Konstantinidou, Stavros Taraviras, Vassilis Pachnis

**Affiliations:** 1 The Francis Crick Institute, Mill Hill Laboratory, The Ridgeway, Mill Hill, London, NW7 1AA UK; 2 Department of Physiology, Medical School, University of Patras, Patras, GR 26 500 Greece; 3 Present address: MRC Clinical Sciences Centre, Imperial College London, Hammersmith Campus, Du Cane Road, London, W12 0NN UK

**Keywords:** Apoptosis, Development, DNA damage, Enteric nervous system, Geminin, Neural crest

## Abstract

**Background:**

In vertebrate organisms, the neural crest (NC) gives rise to multipotential and highly migratory progenitors which are distributed throughout the embryo and generate, among other structures, the peripheral nervous system, including the intrinsic neuroglial networks of the gut, i.e. the enteric nervous system (ENS). The majority of enteric neurons and glia originate from vagal NC-derived progenitors which invade the foregut mesenchyme and migrate rostro-caudally to colonise the entire length of the gut. Although the migratory behaviour of NC cells has been studied extensively, it remains unclear how their properties and response to microenvironment change as they navigate through complex cellular terrains to reach their target embryonic sites.

**Results:**

Using conditional gene inactivation in mice we demonstrate here that the cell cycle-dependent protein Geminin (Gem) is critical for the survival of ENS progenitors in a stage-dependent manner. *Gem* deletion in early ENS progenitors (prior to foregut invasion) resulted in cell-autonomous activation of DNA damage response and p53-dependent apoptosis, leading to severe intestinal aganglionosis. In contrast, ablation of *Gem* shortly after ENS progenitors had invaded the embryonic gut did not result in discernible survival or migratory deficits. In contrast to other developmental systems, we obtained no evidence for a role of Gem in commitment or differentiation of ENS lineages. The stage-dependent resistance of ENS progenitors to mutation-induced genotoxic stress was further supported by the enhanced survival of post gut invasion ENS lineages to γ-irradiation relative to their predecessors.

**Conclusions:**

Our experiments demonstrate that, in mammals, NC-derived ENS lineages are sensitive to genotoxic stress in a stage-specific manner. Following gut invasion, ENS progenitors are distinctly resistant to *Gem* ablation and irradiation in comparison to their pre-enteric counterparts. These studies suggest that the microenvironment of the embryonic gut protects ENS progenitors and their progeny from genotoxic stress.

**Electronic supplementary material:**

The online version of this article (doi:10.1186/s12915-016-0314-x) contains supplementary material, which is available to authorized users.

## Background

During development, stem cells undergo critical changes in their response to dynamic cell-intrinsic programmes or environmental stimuli. Characteristic examples include embryonic stem cells and lineage-specific haemopoietic and neural stem cells, which exhibit distinct responses to gene mutations and environmental stress stimuli depending on their developmental and maturation stage [1–3]. In vertebrates, neural crest (NC) cells constitute a stem cell population which originates at the dorsal neural tube and gives rise to multiple cell types throughout the body [4]. NC cells are highly migratory and travel extensively to reach their final destination in the embryo. Along their migratory routes, NC cells face diverse challenges and are programmed to respond to the unique cellular microenvironments they encounter and adjust to their final homing sites. Despite a good understanding of the cellular, molecular and genetic mechanisms that regulate the development of NC-derived lineages [5], little is known as to how their intrinsic properties change during development and how they respond to the diverse environmental signals they encounter.

NC cells at the vagal level of the neural tube are the primary source of progenitors that generate the enteric nervous system (ENS), the intrinsic neuronal network of the gastrointestinal tract that regulates all aspects of gastrointestinal physiology, including motility, secretion and blood supply [6]. In mice, vagal NC cells emigrate from the neural tube at embryonic day (E) 8.5 and, by E9.0, they have reached the ventrally located anterior branches of the dorsal aorta. Following a ventromedial migratory pathway, a relatively small pool of so-called pre-enteric NC-derived cells (pre-ENCCs) invades the foregut mesenchyme, called hereafter enteric NC-derived cells (ENCCs) [7]. ENCCs migrate rostrocaudally along the gut and proliferate extensively in order to generate sufficient numbers of cells for the timely colonisation of the entire organ [7, 8]. Failure to completely colonise the gastrointestinal tract during embryogenesis leads to Hirschsprung disease (HSCR or congenital megacolon), a congenital enteric neuropathy characterised by absence of enteric ganglia (aganglionosis) from the colon [9].

During embryonic development, ENS lineages pass through diverse cellular terrains, encounter different environmental signals and are likely to change their properties along the way. Consistent with this idea, earlier studies have uncovered stage-specific effects of several loss-of-function mutations on ENCCs. For example, null mutations for the genes encoding the transcription factors Sox10 and Foxd3, which are expressed by early NC cells, lead to severe reduction of pre-ENCCs and total intestinal aganglionosis [10–12]. Similarly, *Phox2b* and *Ret* mutant mice exhibit total absence of the ENS due to elimination of early ENS progenitors [13, 14]. A central phenotypic manifestation of these mutants is the increased apoptotic cell death of NC cells prior or soon after foregut invasion, although the underlying mechanism of this compromised survival remains elusive. Interestingly, following foregut invasion, the impact of some of these mutations on ENCCs is dramatically reduced. For example, conditional ablation of *Foxd3* from NC cells that have already invaded the gut results in a considerably milder ENS deficit which is primarily confined to the most distal segments of the organ [15]. The differential response of pre-ENCCs and ENCCs to loss-of-function gene mutations could be attributed to the respective genes having stage-dependent functions in the ENS lineages. Alternatively, as ENCCs are established within the foregut, they could acquire properties that render them resistant to the deleterious consequences of loss-of-function mutations. This idea is supported by reports demonstrating that, in comparison to other parts of the peripheral and central nervous system (PNS and CNS), apoptotic cell death of ENS cells is rare [16].

To explore the dynamic properties of NC cell lineages and identify potential common mechanisms that underlie their distinct spatiotemporal response to gene mutations, we examined the stage-specific roles of *Geminin* (*Gem*) in ENS development. Gem is a small nuclear protein expressed by all proliferating cells and has been implicated in the maintenance of DNA integrity as well as the self-renewal and commitment of multi-lineage progenitors [17]. In a recent study, we demonstrated that conditional deletion of *Gem* from pre-ENCCs leads to deleterious apoptotic cell death, proliferation deficits and ultimately total intestinal aganglionosis [18]. Nevertheless, the underlying mechanism of these defects remains elusive. In addition, the potential role of Gem in ENCCs that have established themselves within the gut microenvironment is currently unknown.

Here, we demonstrate that Gem is selectively required by pre-ENCCs for genome integrity and cell survival. Deletion of *Gem* from pre-ENCCs results in DNA damage, which is followed by p53-mediated apoptotic cell death. Gem dependence is dramatically diminished as ENCCs invade the gut, indicating a stage-specific requirement of Gem for genome integrity and survival of ENS lineages. We also demonstrate that the stage-specific susceptibility to DNA damage-mediated apoptotic cell death is reproduced by environmental sources of genotoxic stress such as γ-irradiation. Our results provide insight into the mechanisms that promote the survival and fitness of ENS progenitors and highlight the dynamic character of NC lineages as they migrate through the embryo and invade target organs. We suggest that the ENS lineages are protected from cell-intrinsic or environmental sources of genotoxic stress within the gut environment.

## Methods

### Mice

The generation of transgenic and mutant mouse lines used in our studies have been previously described and are as follows: *Gem* ^*FL*^ and *Gem* ^*KO*^ [19], *R26* ^*EYFP*^ [20], *Wnt1Cre* [21], *Sox10iCreER* ^*T2*^ [22], and *p53* null [23].

All animal procedures were performed according to guidelines approved by the UK Home Office under the Animals Act 1986 (Scientific Procedures). Timed matings were set up to generate embryos of defined developmental stage, as indicated in the different studies. The midday of the date on which the vaginal plug was seen was designated as embryonic day 0.5 (E0.5).

### EdU and tamoxifen injections

A 10 mg/mL EdU (Invitrogen, E10187) solution was prepared in PBS. Pregnant females were injected intraperitoneally with 3 μL/g of mouse weight. To study the fraction of cells in the S-phase of the cell cycle, pregnant females were injected 1 hour prior to embryo harvest.

Tamoxifen (Sigma, T5648) was dissolved in a corn oil (Sigma, C8267)-ethanol mixture (9:1) at 25 mg/mL. To induce Cre-mediated recombination in embryos, pregnant females were injected intraperitoneally 35–40 μg/g of mouse weight.

### *In utero* mouse embryo irradiation

Pregnant females at 10.0 and 10.5 day of gestation were subjected to whole-body ionising radiation (IR) using the Gammacell 40 ^137^Caecium Irradiator. Animals were exposed to γ- rays for 1 minute and 19 seconds, which corresponds to a total dose of 1 Gray (Gy). Embryos were harvested 2 and 5 hours following IR and prepared for downstream applications.

### FACS purification, short-term culture of primary ENCCs

Dissected guts from E16.5 embryos were digested with an enzyme mix of 1 mg/mL Dispase/Collagenase (Roche, 296638), at 37 °C. To obtain a cell suspension, culture medium containing 500 μL OptiMEM (Life technologies, 11058-021), 10 % foetal calf serum (FCS, PAA Labs, A15-649), 1 % L-glutamine (Life technologies, 25030-024), 1 % penicillin/streptomycin (Invitrogen, 15140-122), and 0.1 % ciprofloxacin (MP Biomedicals LLC, 199020) antibiotics was added to the digested tissue to aid dissociation with manual pipetting. The suspension was centrifuged at 1000 rpm for 5 minutes at room temperature, the supernatant was discarded and the cell pellet was re-suspended in fresh culture medium (for short-term culture) or simple OptiMEM (for FACS).

For FACS, samples were purified in a FACS ARIA II cell sorter (BD, specialised personnel in Francis Crick Institute, Mill Hill) to isolate YFP^+^ cells. Isolated YFP^+^ cells were collected in OptiMEM and processed for RNA extraction and RT-PCR. For short-term culture, cell suspensions were plated onto Fibronectin (Sigma, F1141)-coated, 8-well Lab-Tek permanox chamber (Thermo Fisher Scientific, 177445) slides. Cultures were then allowed to settle for approximately 15 hours in a 5 % CO_2_ incubator before being fixed and processed for immunofluorescence.

### RT-PCR

Relative quantitation of transcript levels for the genes of our interest was performed using the Taqman Assay-based real-time PCR technology (Life Technologies). The pre-made mixes of fluorescent TaqMan probes and primers used are as follows: b-actin (Mm02619580_g1), BFABP (Mm00445225_m1), Geminin (Mm04206153_g1), HuC (Mm01176703_m1), Phox2b (Mm00435872_m1), Ret (Mm00436304_m1), Sox10 (Mm01300162_m1), YFP (Mr04097229_mr). Reactions were carried out in a 7500 real-time PCR system (Applied Biosystems). Cycle threshold (*Ct*) values for genes of interest were normalised to the *Ct* values of the housekeeping gene b-actin, used as an endogenous control for each cDNA sample. Relative quantitation of transcript levels in the mutant animals compared to the controls was performed by using the 2^–ddCt^ formula.

### Immunohistochemistry, EdU and TUNEL staining

Immunofluorescence was performed on whole-embryo and whole-gut transverse cryosections, on whole-gut preparations and short-term cultures of dissociated embryonic guts. Following permeabilization with PBS containing 0.1–0.3 % Triton X-100 (PBT, Sigma, ×100), a blocking solution that contained 1 % BSA (Sigma, A9647) and 0.15 % glycine (VWR, 01196×) in 0.1 % PBT was added in all types of samples apart from whole-gut preparations, where 10 % Heat-Inactivated Sheep Serum (Biosera, SH650) diluted in 0.3 % PBT was used. Samples were then incubated with primary antibodies (rabbit BFABP (Millipore, ABN14 1:500), rabbit GFP (Thermo Fisher Scientific, A11122 1:1000), rat GFP (Nacalai Tesque, 04404-84 1:500), mouse γH2AX (Millipore, 05-636 1:200), mouse HuC/D (Thermo Fisher Scientific, A21271 1:400), mouse Ki67 (BD Biosciences, 550609 1:200), rabbit pH3 (Millipore, 06-570 1:500), rabbit S100 (Dako, Z0311 1:500), goat Sox10 (Santa Cruz, sc-17343 1:200), and mouse Tuj1 (Covance, MMS-435P 1:1000)) at 4 °C. The following day, samples were washed with PBT and incubated with appropriate fluorophore-conjugated secondary antibodies (all at 1:500, donkey anti-goat alexa 568 (Thermo Fisher Scientific, A11057), donkey anti-mouse alexa 568 (Thermo Fisher Scientific, A10037), donkey anti-rabbit alexa 488 (Thermo Fisher Scientific, A21206), donkey anti-rabbit alexa 568 (Thersmo Fisher Scientific, A10042), donkey anti-rat alexa 488 (Thermo Fisher Scientific, A21208), goat anti-mouse IgG1 alexa 568 (Thermo Fisher Scientific, A21124), goat anti-mouse IgG1 alexa 647 (Thermo Fisher Scientific, A21240), donkey anti-rabbit DyLight^TM^ 405 (Jackson ImmunoResearch, 711-475-152)) for 2 hours at room temperature. This was followed by PBT washes and mounting with Vectashield-DAPI mounting medium.

EdU staining was performed after the completion of the immunofluorescence protocol using the Click-iT EdU Alexa Fluor 594 or 647 Imaging kit (Invitrogen, C10339 or C10340). Similarly, TUNEL was performed after the completion of the immunofluorescence protocol using the ApopTag Red In Situ Apoptosis Detection Kit (Chemicon, S7165).

### Image acquisition and processing

Images of whole guts, gut sections, embryo sections and short-term cultures were taken with the Leica DM6000 confocal microscope fitted with multiphoton spectra physics (SP5) Mai Tai Ti:Sapphire Deep Sea Laser system. The system was run by the Leica Application Suite Advanced Fluorescence (LAS AF) software. Standard excitation and emission filters were used to visualise DAPI, DyLight^TM^ 405, Alexa Fluor 488, Alexa Fluor 568 and Alexa Fluor 647. Each fluorescent image is a projection of a series of z-stacks (2.5 μm thick for whole-gut images, 1–2 μm thick for gut and embryo sections) selected using Fiji and it encompasses almost the entire sample. Adobe Photoshop CS4 was used to adjust image brightness and contrast, as well as for the composition of the final figures.

### Cell counting and integrated density measurements

Quantification of marker expression in short-term cultures was performed under AxioPlan. Gut and embryo cross-section quantifications were performed in single z-stacks per section using the cell counter plugin of Fiji. For gut cross-sections, counting was performed for the stomach, proximal, medial and distal midgut. In order to count equivalent areas of gut in all animal groups compared, caecum and hindgut were not included in the counting as the various mutants exhibited in most of the cases total absence of ENCCs in these parts of the gut. In our embryo cross–section quantifications, counting was performed according to the experiment. In the quantifications aimed for the comparison between the various *Gem* conditional mutants and the respective control animals, representative sections from the rostro-caudal axis of the developing ENS were counted. In E9.5–E10.0 (23–30 somite stage, ss) embryos, when NC cells are still migrating towards the gut, the sections were encompassing the area from the pharynx to the developing gut. In E10.5 and E11.5 embryos, when NC cells have established themselves in the gut, the sections were encompassing the rostrocaudal axis of the gastrointestinal tract (oesophagus to hindgut). In the quantifications aimed for the assessment of IR effect on NC cells that give rise to ENS, counting was performed as described above for the E10.5 embryos. For the E10.0 embryos, counting was focused at the level of the developing gut only and included the cells around the dorsal aorta (pre-ENCCs) and the ENCCs.

In the measurements of integrated density of ENCCs in whole-gut preparations, the GFP staining was used. The measurements were performed in the midgut area only using the appropriate plugins of Fiji. The total length of the midgut was measured and subdivided into four bins of equal length. The integrated density of GFP pixels was measured in the total surface area of each bin, divided by the size of the area (in μm^2^). Final calculations were expressed as integrated density per μm^2^ for each bin.

### Statistical analysis

In all experiments, at least three embryos that came from more than one litter were analysed (see Additional file 1: raw data for further details on the number of samples used). Data were expressed as mean ± standard error of the mean (SEM). No samples were excluded from the statistical analyses. In all the statistical analyses performed, we did not assume equal standard deviation amongst datasets. In more detail, we used (1) multiple t-tests without correction for multiple comparisons when two datasets were compared for multiple parameters, (2) unpaired t-test with Welch’s correction when two datasets were compared for one parameter, (3) paired t-test to compare the pre-ENCCs and ENCCs within the same animal, (4) one-way ANOVA followed by uncorrected Fisher’s LSD multiple comparison test when more than two datasets were compared with each other, and (5) two-way ANOVA followed by uncorrected Fisher’s LSD multiple comparisons test in the measurements of ENCC integrated density in order to take into consideration both the genotype of the animals and the midgut bin. Histograms and statistical analysis were all performed in GraphPad Prism version 5.

## Results

### Ablation of *Gem* from pre-ENCCs results in activation of DNA damage response, cell cycle deficits and apoptosis

Our previous studies have shown that deletion of *Gem* from early NC cells results in almost complete intestinal aganglionosis due to proliferation defects and apoptosis of pre-ENCCs [18]. To explore the mechanisms by which *Gem* deletion compromises the proliferation and survival of pre-ENCCs, we combined the *Gem* ^*FL*^ (conditional knock-out) and *Gem* ^*KO*^ (null) alleles [19] with the *Wnt1Cre* transgene, which drives expression of Cre recombinase in early NC cells [21] and the Cre-dependent *R26* ^*EYFP*^ reporter [20] to mark the targeted cells. As expected, the ENS phenotype of *Wnt1Cre;Gem* ^*FL/KO*^ *;R26EYFP* (hereafter designated as *Wnt1Cre|Gem*) relative to *Wnt1Cre;Gem* ^*FL/+*^ *;R26EYFP* (control) embryos was similar to that reported in our previous study [18] (Additional file 2: Figure S1A, B). Nevertheless, some of the *Wnt1Cre|Gem* embryos analysed in the current study showed a somewhat milder aganglionosis (Additional file 2: Figure S1C), which may relate to the drift of the mixed genetic background.

**Fig. 1:**
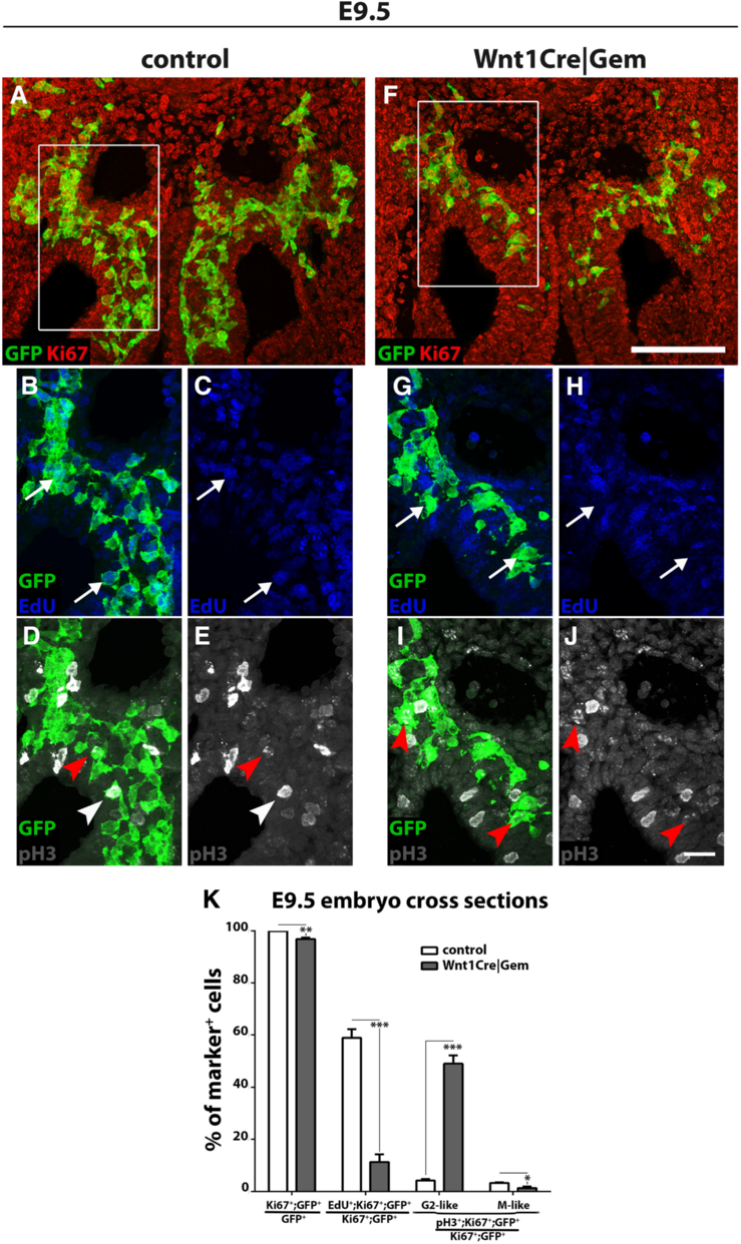
Ablation of *Gem* from early pre-ENCCs results in cell cycle deficits. Cryosections through the foregut area of control (**a**–**e**) and *Wnt1Cre|Gem* (**f**–**j**) E9.5 embryos immunostained for GFP (green), Ki67 (red) and pH3 (white), and processed for EdU labelling (blue). **b**–**e** is an enlarged view of the area boxed in **a. g**–**j** is an enlarged view of the area boxed in **f**. White arrows in **b**, **c**, **g** and **h** indicate GFP^+^ NC cells positive or not for EdU. Red and white arrowheads in **d**, **e**, **i** and **j** indicate GFP^+^ NC cells with a G2-like and M-like pH3 staining, respectively. **k, ** quantification of NC (GFP^+^) cells that are positive for Ki67, proliferating (Ki67^+^;GFP^+^) NC cells that are in S-phase (EdU^+^) or in the G2 or M-phase (pH3^+^) of the cell cycle. Multiple t-tests without correction, *P_value_ < 0.05, **P_value_ < 0.01, ***P_value_ < 0.001. Scale bars: (**a**, **f**) 100 μm, (**b**–**e** and **g**–**j**) 25 μm

First, we determined the cell cycle distribution of *Gem*-deficient pre-ENCCs. For this, cryosections from E9.5 control and *Wnt1Cre|Gem* embryos were immunostained for Ki67 (to label all cycling cells) [24], pH3 (to mark cells in G2-M) [25] and YFP, and assessed for EdU incorporation (to label cells in S-phase) [26]. For pH3, we also analysed its nuclear distribution pattern, which distinguishes between the G2 and M phases of the cell cycle. Specifically, during the G2 phase, pH3 shows a spotted pattern (G2-like), whereas mitotic cells show a more ubiquitous nuclear staining resulting from chromosome condensation (M-like) [27]. Relative to controls, *Wnt1Cre|Gem* embryos showed a small but statistically significant reduction in the fraction of Ki67^+^ NC cells (Fig. 1a, f, k). Interestingly, the percentage of Ki67^+^ NC cells exhibiting a G2-type of pH3 staining pattern was dramatically increased in *Wnt1Cre|Gem* animals, while Ki67^+^ NC cells with M-type of pH3 staining pattern were under-represented in these embryos (Fig. 1d, e, i–k). Finally, we also observed that the fraction of EdU^+^ NC cells was dramatically reduced in *Wnt1Cre|Gem* embryos relative to controls (Fig. 1b, c, g, h, k). Taken together, our molecular marker analysis suggests that *Gem*-deficient early ENS progenitors are characterised by cell cycle deficits, while the nuclear pH3 staining pattern is consistent with G2 phase arrest.

**Fig. 2:**
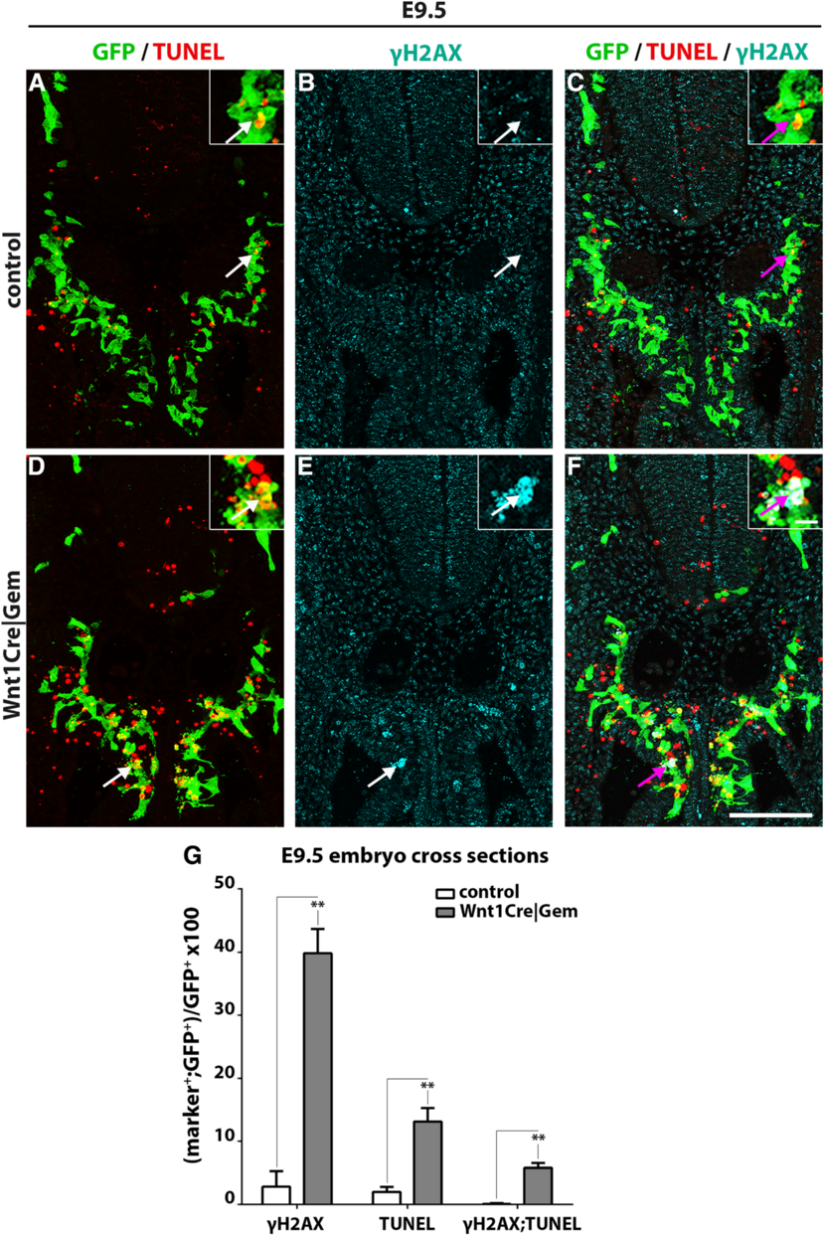
Ablation of *Gem *from early pre-ENCCs results in robust DNA damage response and apoptotic cell death. Cryosections at the level of the foregut of control (**a**–**c**) and *Wnt1Cre|Gem* (**d**–**f**) E9.5 embryos immunostained for GFP (green), γH2AX (cyan) and processed for TUNEL (red). Insets correspond to the area in the panels indicated with arrows. **g, ** Quantification of NC (GFP^+^) cells that have DNA damage (γH2AX^+^), undergo apoptosis (TUNEL^+^) or both (γH2AX^+^;TUNEL^+^). Multiple t-tests without correction, **P_value_ < 0.01. Scale bars: (**a**–**f**) 100 μm, (insets) 10 μm

G2 phase arrest and failure to enter mitosis have been associated with defects in DNA replication and activation of the DNA damage response pathway [28, 29]. Given the role of Gem in DNA replication and genome integrity reported for other lineages and cells in culture [30–32], we considered the possibility that the apoptosis and associated developmental defects observed in the ENS of *Wnt1Cre|Gem* embryos result from the activation of the DNA damage response. To examine this possibility, cryosections from E9.5 control and *Wnt1Cre|Gem* embryos were double immunostained for YFP and γH2AX, a modified histone used widely as a surrogate marker of DNA damage [33], and processed them in parallel for TUNEL to visualise apoptotic cells [34]. Relative to controls, *Wnt1Cre|Gem* embryos showed a dramatic increase in the fraction of γH2AX^+^ and TUNEL^+^ pre-ENCCs and early ENCCs that have just invaded the foregut (Fig. 2a, b, d, e, g). Moreover, a significant proportion of the γH2AX^+^ pre-ENCCs and ENCCs were also positive for TUNEL (Fig. 2c, f, g), indicating that, in *Wnt1Cre|Gem* embryos, NC cells with DNA damage underwent apoptotic cell death. In further support for DNA damage in NC cells of *Wnt1Cre|Gem* embryos, a considerable fraction of γH2AX^+^;YFP^+^ cells contained abnormally large nuclei (Additional file 3: Figure S2D–F). Such “giant nuclei” have been reported previously in *Gem* loss-of-function studies in flies and human cell lines and are thought to result from genome over-replication [35, 36]. Consistent with the extensive apoptosis observed at E9.5, analysis of sections from E10.5 and E12.5 embryos showed a dramatic reduction of YFP^+^ cells in the gut of *Wnt1Cre|Gem* embryos and almost complete absence of γH2AX^+^ or TUNEL^+^ ENCCs, suggesting that the majority of *Gem*-deficient ENCCs had been eliminated by this stage (Additional file 4: Figure S3). Occasionally, we observed that pre-ENCCs and ENCCs of E10.5 *Wnt1Cre|Gem* embryos appeared to be more clustered relative to controls (Additional file 4: Figure S3D–F and Fig. 1). This phenotype, however, was not observed at later developmental stages (Additional file 4: Figure S3K–M and Fig. 4 k-m). Taken together, these experiments suggest that deletion of *Gem* from pre-ENCCs leads to cell cycle defects and activation of DNA damage response leading ultimately to apoptotic cell death.

### *p53* deletion rescues the aganglionosis phenotype of *Wnt1Cre|Gem* embryos

**Fig. 3:**
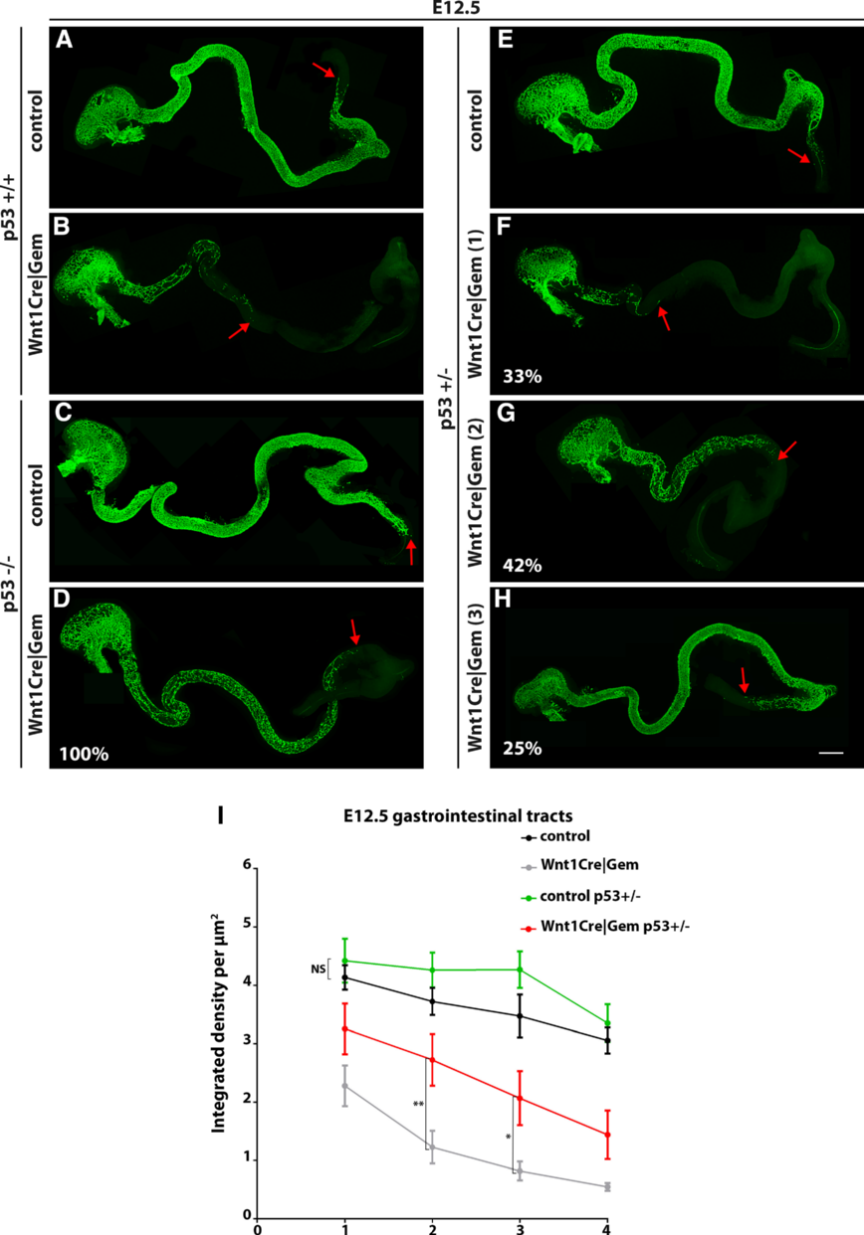
Ablation of *p53* rescues the intestinal aganglionosis of *Wnt1Cre|Gem* embryos. Whole-mount gut preparations of control (**a**), control *p53* ^*+/–*^ (**e**), control *p53* ^*–/–*^ (**c**), *Wnt1Cre|Gem* (**b**), *Wnt1Cre|Gem p53* ^*+/–*^ (**f**–**h**) and *Wnt1Cre|Gem p53* ^*–/–*^ (**d**) E12.5 embryos immunostained for GFP to visualise migrating ENCCs. Red arrows indicate the position of the most caudally located ENCCs in the gut preparations. Percentages in **d**, **f**, **g** and **h** represent a quantification of the analysed gut preparations that show the phenotype depicted in these panels. **i, **Quantification of the integrated density of GFP^+^ ENCCs per μm^2^ in the midgut of control and mutant embryos divided in four equal length bins. Two-way ANOVA followed by Fisher’s LSD multiple comparison test, *P_value_ < 0.05, **P_value_ < 0.01, NS: non-significant. Scale bar: (**a**–**h**) 400 μm

In several cell lineages, DNA damage-induced apoptosis depends on the tumour suppressor protein p53 [1, 2]. To determine whether the apoptotic cell death of *Gem*-deficient ENS progenitors requires p53 activity, we examined the effect of *p53* deletion [23] on the colonisation of *Wnt1Cre|Gem* embryo gut by ENCCs at E12.5, a stage at which the front of migrating NC cells is normally within the colon. *p53* heterozygosity *per se* had no adverse effects on gut colonisation by NC cells and ENS development (Fig. 3a, e, i). In contrast, *Wnt1Cre|Gem* embryos heterozygous for *p53* deletion (*Wnt1Cre|Gem p53* ^*+/–*^) showed significant rescue of the colonization deficit (Fig. 3f–i). Specifically, out of 12 *Wnt1Cre|Gem p53* ^*+/–*^ embryos, four (i.e. 33 %) showed no improvement relative to *Wnt1Cre|Gem* embryos (Fig. 3f), five (i.e. 42 %) showed significant improvement (Fig. 3g) and three (i.e. 25 %) were indistinguishable from control (*p53* ^*+/–*^) animals (Fig. 3h). These results were corroborated by analysis of five E12.5 *Wnt1Cre|Gem p53* ^*–/–*^ embryos in all of which the migratory front of ENCCs was located at the ileocecal valve, clearly ahead of the average position of the migratory front in similar stage *Wnt1|Gem* embryos (Fig. 3c, d). Taken together, these studies support the idea that the intestinal aganglionosis observed in *Wnt1Cre|Gem* embryos is largely due to p53-dependent apoptotic cell death.

**Fig. 4:**
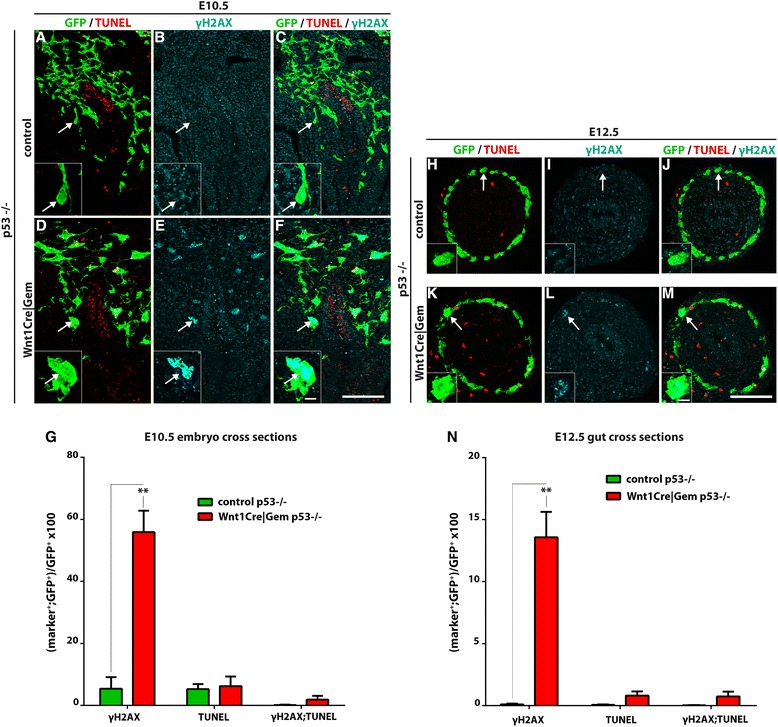
Ablation of *p53* leads to accumulation of DNA damage in *Wnt1Cre|Gem* embryos. E10.5 embryo cryosections at the level of the stomach (**a**–**f**) and E12.5 gut cryosections at the level of the midgut (**h**–**m**) of control *p53* ^*–/–*^ (**a**–**c** and **h**–**j**) and *Wnt1Cre|Gem p53* ^*–/*–^ (**d**–**f** and **k**–**m**), immunostained for GFP (green), γH2AX (cyan) and processed for TUNEL (red). Insets correspond to the areas in the panels indicated with arrows. **g**, **n, ** Quantification of NC (GFP^+^) cells that have DNA damage (γH2AX^+^), undergo apoptosis (TUNEL^+^) or both (γH2AX^+^;TUNEL^+^). Multiple t-tests without correction, **P_value_ < 0.01. Scale bars: (**a**–**f**, **h**–**m**) 100 μm, (insets) 10 μm

If apoptosis of pre-ENCCs in *Wnt1Cre|Gem* embryos is due to DNA damage, we expected that inhibition of p53 would lead to accumulation of γH2AX^+^ cells. Indeed, we observed a dramatic increase in the fraction of γH2AX^+^;YFP^+^ cells (many of which had enlarged nuclei) in the gut of E10.5 *Wnt1Cre|Gem p53* ^*–/–*^ embryos relative to control *p53* ^*–/–*^ littermates (Fig. 4b, e, g). In contrast, the percentage of TUNEL^+^ cells was similar between the two genotypes (Fig. 4a, c, d, f, g). At E12.5, γH2AX^+^ NC cells were still prevalent in the gut of *Wnt1Cre|Gem p53* ^*–/–*^ embryos, but the fraction of TUNEL^+^;YFP^+^ cells was not significantly different between the two groups of animals (Fig. 4 h–n). Taken together, our results demonstrate that deletion of *Gem* from early pre-ENCCs results in activation of DNA damage response, which leads to p53-mediated apoptotic cell death and is the main cause of the observed intestinal aganglionosis.

### No evidence for a direct role of Gem in the commitment of ENS progenitors

**Fig. 5:**
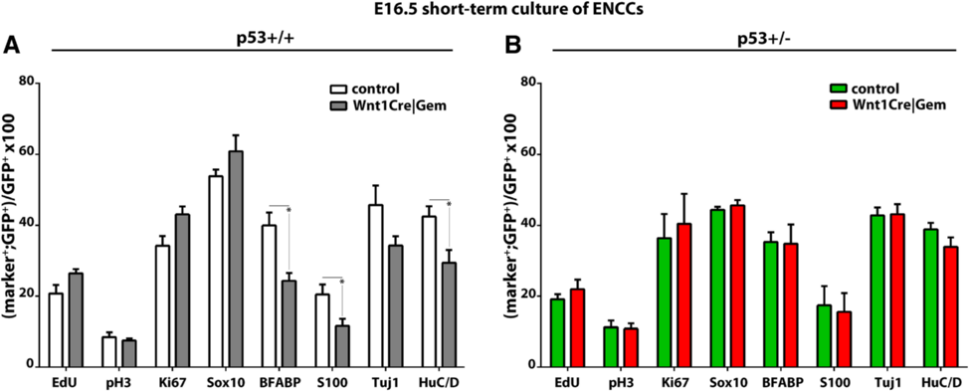
Apparent lineage commitment deficits of ENCCs from *Wnt1Cre|Gem* embryos are rescued by *p53* mutation. Short-term cultures of GFP^+^ ENCCs from control and *Wnt1Cre|Gem* embryos (**a**) and control *p53* ^*+/–*^ and *Wnt1Cre|Gem p53* ^*+/–*^ embryos (**b**), immunostained for GFP, Ki67, pH3, Sox10, BFABP, S100, Tuj1 and HuC/D, and processed for EdU labelling. Multiple t-tests without correction, *P_value_ < 0.05

Previous reports have suggested that Gem is implicated in the commitment and differentiation of diverse lineages [37, 38]. To explore whether Gem also controls commitment and differentiation of ENS precursors, we compared the expression of progenitor and lineage markers in short-term cultures of dissociated E16.5 guts from control and *Wnt1Cre|Gem* embryos. Specifically, we analysed the expression of Sox10, a marker of ENS progenitors and enteric glia [39], BFABP and S100, which specifically mark enteric glial cells [39], and Tuj1 and HuC/D to label enteric neurons [40, 41]. While the fraction of Sox10^+^ cells amongst the YFP^+^ cell population was similar in the two types of cultures, the representation of enteric neurons and glia was reduced in *Wnt1Cre|Gem* cultures (Fig. 5a). The reduced number of ENS cells is unlikely to result from reduced proliferation of neuronal and glial progenitors since we recorded no difference in the percentage of EdU^+^, pH3^+^ and Ki67^+^ ENCCs between the two types of cultures (Fig. 5a).

To determine whether the apparent differentiation deficit in *Wnt1Cre|Gem* cultures was related to the pro-apoptotic effect of *Gem* deletion, we took advantage of the observation that *p53* heterozygosity rescues the apoptosis of *Gem*-deficient cells. Remarkably, no difference was detected in the percentages of the neuronal and glial populations between control *p53* ^*+/–*^ and *Wnt1Cre|Gem p53* ^*+/–*^ embryos (Fig. 5b). Together, our studies suggest that Gem is required for genome integrity, cell cycle regulation and survival of early ENS progenitors but provide no evidence of a direct effect of *Gem* deletion on ENCC commitment and differentiation.

### Stage-specific effects of *Gem* deletion on genome integrity and survival of the ENS lineages

**Fig. 6:**
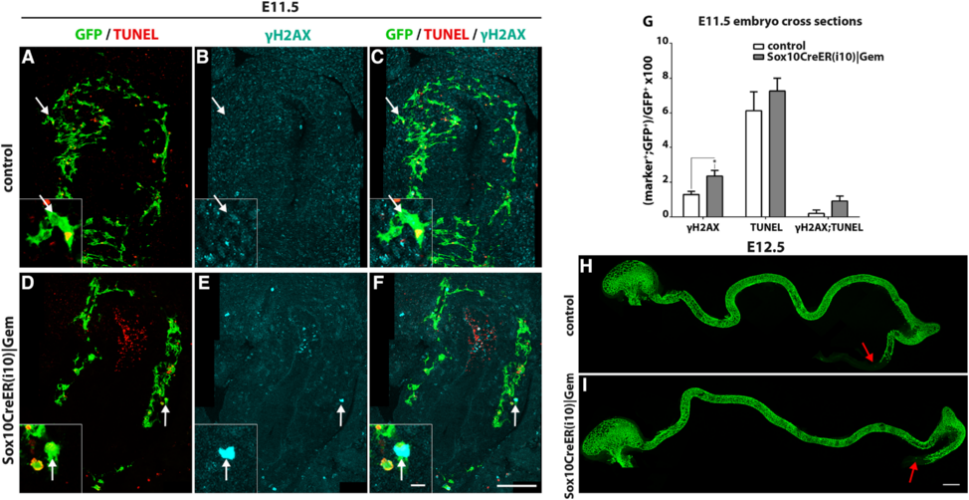
Ablation of *Gem* from ENCCs leads to low level DNA damage and largely normal gut colonisation. Cryosections at the level of the stomach of control (**a**–**c**) and *Sox10CreER(i10)|Gem* (**d**–**f**) E11.5 embryos, immunostained for GFP (green), γH2AX (cyan) and processed for TUNEL (red). Insets correspond to the areas in the panels indicated with arrows. **g,** Quantification of NC (GFP^+^) cells that have DNA damage (γH2AX^+^), undergo apoptosis (TUNEL^+^) or both (γH2AX^+^;TUNEL^+^). Multiple t-tests without correction, *P_value_ < 0.05. Whole-mount gut preparations of control (**h**) and *Sox10CreER(i10)|Gem* (**i**) E12.5 embryos immunostained for GFP to visualise the distribution of ENCCs along the gut. Red arrows indicate the position of the most caudally located ENCCs in the gut preparations. Scale bars: (**a**–**f**) 100 μm, (**h**–**i**) 400 μm, (insets) 10 μm

Several stem cell lineages (including neural and hematopoietic stem cells) show a stage-specific susceptibility to genotoxic stress, which may be related to cell-intrinsic changes or changes in the microenvironment [1, 3]. To determine whether NC cell lineages show stage-dependent susceptibility to DNA damage, we compared the consequences of NC-specific deletion of *Gem* induced at E8.5 (a stage at which most of ENS progenitors have not invaded the foregut) or E10.0 (a stage that which both pre-ENCCs and ENCCs can be identified). For this experiment, we combined the *Gem* ^*FL*^ and *Gem* ^*KO*^ alleles with the tamoxifen-inducible NC-specific *Sox10iCreER* ^*T2*^ transgene [22] and the *R26* ^*EYFP*^ reporter to generate *Sox10iCreER* ^*T2*^ *;Gem* ^*FL/+*^ *;R26* ^*EYFP*^ (control) and *Sox10iCreER* ^*T2*^ *;Gem* ^*FL/KO*^ *;R26EYFP* (*Sox10CreER|Gem*) embryos. Exposure of E8.5 *Sox10CreER|Gem* embryos to tamoxifen largely reproduced the colonisation defect observed in *Wnt1Cre|Gem* animals (Additional file 5: Figure S4B). Nevertheless, the aganglionosis of *Sox10CreER(i8.5)|Gem* embryos was somewhat milder relative to that observed in *Wnt1Cre|Gem* embryos. This may result from the differential kinetics of *Gem* deletion induced by the *Wnt1Cre* and *Sox10iCreER* ^*T2*^ transgenes, the latter requiring 12–24 hours to reach maximum activity following tamoxifen administration [42], suggesting a stage-specific effect of *Gem* deletion on ENS development. In support of this view, exposure of *Sox10CreER|Gem* embryos to tamoxifen 1½ days later (at E10.0) resulted in no obvious defects in gut colonisation by YFP^+^ cells (Fig. 6 h, i), despite efficient ablation of *Gem* in these cells (Additional file 5: Figure S4D). γH2AX immunostaining in combination with TUNEL on embryo cryosections at E11.5 showed a modest but statistically significant increase in the fraction of γH2AX^+^;YFP^+^ cells in *Sox10CreER|Gem* embryos induced at E10.0, which was not associated with increased apoptosis (Fig. 6a–g). Finally, ENCCs from *Sox10CreER|Gem* embryos induced at E10.0 did not show obvious deficits in neuronal or glial differentiation (Additional file 6: Figure S5).

To examine whether the stage-specific response of ENCCs to *Gem* ablation is related to changes in their proliferative capacity, we counted the proliferating ENCCs at E9.5 and E11.5, time points which mark the end of the period Cre recombinase was expected to be active in following tamoxifen administration at E8.5 (or driven by the constitutive transgene *Wnt1Cre*) or E10.0, respectively [21, 42]. Immunostaining for Ki67 was examined on embryo sections from *Wnt1Cre|EYFP* transgenic embryos in which ENCCS and their derivatives were marked by YFP. Our analysis revealed a modest yet insignificant reduction of cycling (Ki67^+^) YFP^+^ cells at E11.5 relative to E9.5 embryos (Additional file 7: Figure S6A–C). This reduction is at least partly due to increased representation of post mitotic neurons among the YFP^+^ cells present in the gut of E11.5 embryos [39]. Nevertheless, this relatively small change was unlikely to account for the dramatic difference (~17-fold) in the percentage of γH2AX^+^ cells within the population of YFP^+^ cells in the gut of *Sox10CreER(i10)|Gem* and *Wnt1Cre|Gem* embryos (Additional file 7: Figure S6D). Taken together, our data indicate that *Gem* deletion induces less extensive DNA damage response and apoptosis in ENCCs relative to their pre-ENCC predecessors and that *Gem* function is largely dispensable for survival and genome integrity following entry of ENS progenitors into the gut.

### Stage-specific response of ENS lineages to environmental sources of genotoxic stress

**Fig. 7:**
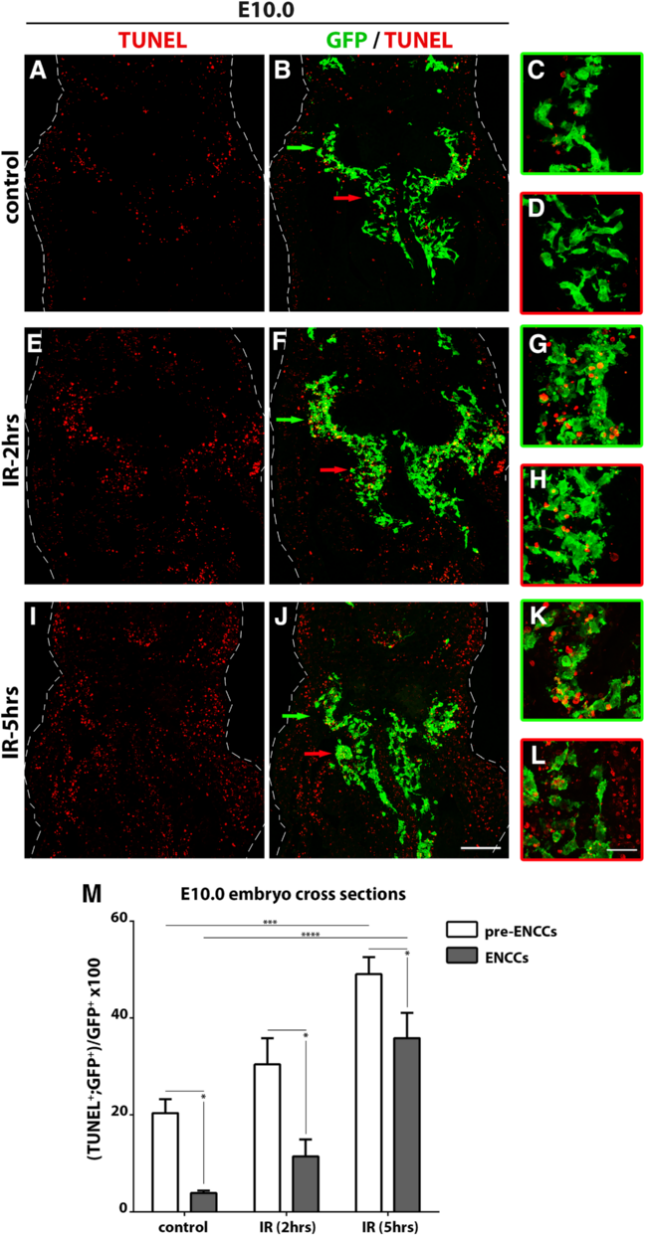
Differential sensitivity  of pre-ENCCs and ENCCs to γ-irradiation. Cryosections at the level of the foregut of E10.0 *Wnt1Cre|EYFP* control embryos (**a**–**d**), *Wnt1Cre|EYFP* embryos 2 hours (**e**–**h**), or 5 hours (**i**–**l**) following exposure to IR, immunostained for GFP (green) and processed for TUNEL (red). Green and red arrows indicate the area of pre-ENCCs and ENCCs, respectively, magnified within a colour-matched frame. **m,** Quantification of GFP^+^ pre-ENCCs and ENCCs that are undergoing apoptosis (TUNEL^+^). Paired t-test between pre-ENCCs and ENCCs within each group, *P_value_ < 0.05. One-way ANOVA followed by Fisher’s LSD multiple comparison test between the pre-ENCCs or the ENCCs of the three groups, ***P_value_ < 0.001, ****P_value_ < 0.0001. Scale bars: (**a**–**b**, **e**–**f**, **i**–**j**) 100 μm, (**c**–**d**, **g**–**h**, **k**–**l**) 25 μm

To examine whether the stage-specific sensitivity of ENCCs to *Gem* ablation applies to other unrelated sources of genotoxic stress, we exposed mouse embryos *in utero* to ^137^Caesium-emitted IR (γ-rays), which is known to upregulate γH2AX in cultured cells [33] and induces apoptotic cell death of CNS neural precursors in vivo [43]. Pilot experiments showed that 1 Gy of IR is sufficient to induce widespread expression of the γH2AX marker in mouse embryos. Embryos were irradiated at E10.0, a stage at which both pre-ENCCs and ENCCs co-exist, allowing a direct comparison of their response to IR-induced genotoxicity, and at E10.5, when the founding population of ENS precursors has fully invaded the gut mesenchyme. *Wnt1Cre|EYFP* embryos were exposed to γ-rays (1 Gy) and harvested 2 or 5 hours later. At E10.0, non-irradiated control animals failed to upregulate γH2AX expression (Additional file 8: Figure S7A, B). However, 2 hours after IR, embryos showed a dramatic upregulation of γH2AX expression in multiple lineages, consistent with efficient induction of DNA damage (Additional file 8: Figure S7C, D). Next, we specifically assessed the apoptotic response of pre-ENCCs and ENCCs to IR-induced genotoxicity. In control sections from non-irradiated embryos we observed a relatively small fraction of cells undergoing apoptotic cell death mostly among the population of pre-ENCCs (Fig. 7a–d, m). However, 2 or 5 hours after IR, the apoptotic response of YFP^+^ cells was dramatically increased, but remarkably, ENCCs within the gut wall showed increased resistance to γ-ray-induced cell death (Fig. 7e–m). The relative resistance of NC cells in the gut to IR-mediated apoptotic cell death was further confirmed at E10.5; 2 hours after IR the percentage of TUNEL^+^ ENCCs remained largely unchanged (Additional file 9: Figure S8A–D, G). As expected, 5 hours after IR, apoptosis was widespread across the whole embryo section and the NC apoptotic cell fraction had increased (Additional file 9: Figure S8E–G). In summary, we have shown that NC cells that give rise to the ENS have a stage-specific sensitivity to DNA damage-induced apoptotic cell death. Pre-ENCCs are sensitive to IR-induced apoptotic cell death while ENCCs are relatively resistant, at least during the immediate postIR period (i.e. < 5 hours).

## Discussion

The central role of Gem in safeguarding genome integrity during cell cycle progression is well documented. However, most studies so far have been carried out in cultured cell lines [36, 44], using flies or frogs as model organisms [30, 35]. Recently, a study by Barry et al. [45] has reported that Gem is required for genome integrity of mouse spermatogonia, which upon deletion of *Gem*, accumulate DNA damage, fail to enter mitosis and are subsequently eliminated. Here, we provide evidence that Gem activity is also required during mammalian embryogenesis to maintain the genomic integrity of NC-derived ENS lineages. Our data support a model according to which *Gem* deletion leads to robust DNA damage response and arrest at the G2 phase of the cell cycle followed by apoptotic cell death. These responses were temporally regulated in that DNA damage and cell death were observed in pre-ENCCs while NC cells within the gut (ENCCs) were relatively resistant to *Gem* ablation. Our experiments suggest that, in mice, DNA damage in response to *Gem* deletion is more widespread than previously appreciated and emphasise the importance of systematic analysis of the effects of *Gem* ablation in other mammalian cell lineages.

In addition to its roles in DNA replication, Gem is thought to control the self-renewal and commitment of progenitor populations across different lineages of mice, including embryonic, haemopoietic and neural stem cells [19, 37, 38]. In an apparent agreement with these reports, deletion of *Gem* from pre-ENCCs resulted in deficits in enteric neuron and glial differentiation. However, introduction of a *p53* mutation into the *Gem-*deficient genetic background restored both deficits, indicating that Gem does not have a direct role in the commitment or differentiation of ENS lineages. In further support of this idea, we failed to detect any changes in the proportion of enteric neurons and glia when *Gem* was deleted from ENCCs, which showed no major DNA damage response or apoptosis. Taken together, these studies argue that the main role of Gem in NC lineages is to control DNA replication, cell cycle and genomic DNA integrity.

The stage-specific requirement of Gem identified here is unlikely to be related to reduced proliferative capacity of ENCCs in comparison to their pre-ENCC counterparts, since similar rates of proliferation were detected for both cell types (Additional file 7: Figure S6). Alternatively, it is possible that other molecular mechanisms known to safeguard DNA replication fidelity in metazoa, such as the proteolytic degradation of Cdt1 and the Cdk-dependent phosphorylation of pre-replicative complex components [46], could specifically operate in ENCCs. In support of this possibility, Cdt1 stabilisation is known to worsen the genomic instability phenotype resulting from *Gem* deletion [31]. Further genetic studies will be necessary to examine the relative contribution of these pathways in maintaining genomic stability during ENS lineage progression. The relative resistance of ENCCs to *Gem* deletion could also be explained by the effects of the gut microenvironment, which could activate within ENCCs non-specific mechanisms that protect them from genotoxic stress. In support of this idea, exposure of pre-ENCCs and ENCCs to exogenous γ-irradiation reproduced the stage-specific effects of *Gem* ablation in that, relative to ENCCs, pre-ENCCs were markedly more sensitive to apoptotic cell death in response to DNA damage. Indeed, we observed a gradient of TUNEL^+^ signal in the foregut of irradiated embryos with ENCCs deep in the foregut less likely to undergo apoptosis (Fig. 7). This is reminiscent of the situation in chick embryos in which the small number of apoptotic ENCCs observed under physiological conditions have either not entered the foregut or are restricted to its most proximal part [47]. Interestingly, the stage-specific effects of *Gem* ablation and γ-irradiation are reproduced by other mutations affecting ENS development. Thus, deletion of key ENS regulators in pre-ENCCs leads to almost complete elimination of these cells by apoptosis [10–14], while similar mutations introduced shortly after ENS progenitors invade the gut resulted in significantly milder phenotypes [15, 48]. Together, our current findings and previous genetic studies highlight a more general property of ENCCs, namely their ability to resist the adverse effects of gene mutations or genotoxic stress when they reside within the microenvironment of the gut wall.

The cellular and molecular mechanisms underpinning the ability of NC cells to resist the potential detrimental effects of genotoxic stress are currently unknown. Nevertheless, potential clues could be provided by the specific molecular signals encountered by pre-ENCCs as they take residence within the gut wall. One such signal is the neurotrophic factor Gdnf, which is specifically expressed by the gut mesenchyme and activates the receptor tyrosine kinase Ret (induced in pre-ENCCs as they approach the foregut) to promote ENCC survival and proliferation [40, 49]. Based on the spatiotemporal pattern of Ret and Gdnf expression and their documented role in promoting the survival of ENS lineages, we suggest that activation of this signalling pathway may provide non-specific pro-survival signals to ENCCs. Interestingly, a recent study has shown that Ret transduces survival signals in haemopoietic stem cells and protects their reconstitution potential upon transplantation [50], suggesting that the pro-survival role of Ret may be more general than currently appreciated. Furthermore, activation of a closely related member of the receptor tyrosine kinase superfamily, Fgfr, is thought to mediate enhanced resistance of human keratinocytes to γ-rays by activating DNA repair mechanisms [51]. The identification of receptor tyrosine kinase signalling pathways as mediators of DNA repair and cell survival [51–53] raises the possibility that a similar scenario is true in the developing ENS. In particular, it is possible that Ret, in addition to enhancing ENCC survival, also activates DNA repair mechanisms within ENCCs, thus contributing to the minimal DNA damage observed upon deletion of *Gem* from ENCCs. The role of Ret in the pathogenesis of most (if not all) cases of HSCR is well-established. However, by highlighting the importance of genome integrity for ENS development, our current studies raise the possibility that apoptotic cell death in response to DNA damage constitutes a novel pathogenetic mechanism of intestinal aganglionosis and HSCR. This suggestion is supported by a recent pathway-based computational study which implicated novel loci associated with genome stability in the pathogenesis of HSCR [54].

Stage-specific response of progenitor cell populations to cell-intrinsic or environmental genotoxic stress has been described previously in the nervous system. Thus, deletion of the gene encoding the DNA replication component TopBP1 from the forebrain of E10.0 mouse embryos resulted in DNA damage-induced apoptosis of early neural progenitors. In contrast, efficient ablation of this locus 24 hours later led to a much milder phenotype indicating a relative resistance of these cells to the genotoxic effects of *TopBP1* deletion [1]. Furthermore, mouse pluripotent epiblast cells of the early mouse embryo are particularly sensitive to genotoxic stress after exposure to IR as opposed to their differentiated progeny [55]. These studies highlight the low tolerance of early stem cell populations to harmful genotoxic agents that could potentially compromise their downstream lineages. We suggest that the enhanced sensitivity of pre-ENCCs to genotoxic agents ensures the fitness of the initial pool of ENS progenitors that will ultimately generate the vast network of enteric neurons and glia that are essential for maintaining organismal survival and homeostasis.

However, what is the advantage of ENS lineages (and other neuroectodermal lineages) ultimately becoming resistant to DNA damage? A potential explanation could be provided by the recent discovery that DNA damage is a natural consequence of normal brain activity [56]. Since spontaneous activity of enteric neurons has also been detected in mouse embryos [57], it is possible that molecular mechanisms safeguarding DNA integrity must be in place as ENS progenitors start to differentiate and generate functional enteric neurons. Although the significance of DNA integrity in adult ENS is unexplored, acquired ENS deficits during adulthood could be associated with deterioration of genome protection mechanisms. Ageing is a characteristic example of ENS dysfunction, which is observed in the elderly population and is associated primarily with reduced enteric neuron numbers and neuronal malfunction [58]. At present, many different mechanisms of enteric neuronal cell loss during ageing have been suggested. Of these, cellular senescence of enteric neurons is induced by a DNA damage response [59].

## Conclusions

The progenitors of the ENS show stage-specific response to genotoxic insults associated with a gene mutation and an environmental DNA damaging agent. Deletion of the cell cycle regulator *Gem* from the pre-ENCCs results in abrogation of the ENS progenitor population and severe intestinal aganglionosis as opposed to deletion of the same gene following gut entrance. The underlying mechanism of this detrimental phenotype is the activation of DNA damage signalling followed by p53-mediated apoptotic cell death. Finallly, pre-ENCCs and ENCCs show differential sensitivity to γ-irradiation. Although both populations activate a DNA damage response it is primarily the pre-ENCC population that dies apoptotically.

Our findings highlight previously unappreciated stage-specific properties of ENS progenitors and give valuable hints as to how gut environment confers an advantage to the developing and adult neuronal network. Gaining insight into the underlying mechanisms that mediate the sensitivity and subsequent resilience of the embryonic ENS population will offer valuable information for the potential causes of congenital neuropathies, such as HSCR, but also of progressive deterioration in gut function observed in aging and acquired neurodegenerative disorders.

## Additional files

Additional file 1: Raw data. (TIF 482 kb)
Additional file 2:
**Figure S1.** Ablation of *Gem* from early pre-ENCCs leads to severe intestinal aganglionosis. Whole-mount gut preparations of control (A) and *Wnt1Cre|Gem* (B–C) E12.5 embryos immunostained for GFP to visualise migrating ENCCs. Red arrows indicate the position of the most caudally located ENCCs in the gut preparations. Scale bar: (A–C) 400 μm. (TIF 2895 kb)
Additional file 3:
**Figure S2.** Ablation of *Gem* from early pre-ENCCs leads to DNA damage associated with enlargement of the affected nuclei. Cryosections at the level of the foregut of control (A–C) and *Wnt1Cre|Gem* (D–F) E9.5 embryos, immunostained for GFP (green), γH2AX (cyan), processed for TUNEL (red) and counterstained with DAPI (grey). White arrows and yellow dotted line mark the enlarged nucleus of DNA damaged NC cells that are not undergoing apoptosis. Scale bar: (A–F) 20 μm. (TIF 6057 kb)
Additional file 4:
**Figure S3.** Ablation of *Gem* from early pre-ENCCs results in elimination of DNA-damaged and apoptotic cells. Embryo cryosections at the level of the stomach and gut cryosections at the level of the midgut of control (A–C and H–J) and *Wnt1Cre|Gem* (D–F and K–M) E10.5 and E12.5 embryos, respectively, immunostained for GFP (green), γH2AX (cyan) and processed for TUNEL (red). (G, N) Quantification of NC (GFP^+^) cells that have DNA damage (γH2AX^+^), undergo apoptosis (TUNEL^+^) or both (γH2AX^+^;TUNEL^+^). Insets correspond to the areas in the panels indicated with arrows. Multiple t-tests without correction, insignificant differences observed. Scale bars: (A–F, H–M) 100 μm, (insets) 10 μm. (TIF 702 kb)
Additional file 5:
**Figure S4.** Efficient ablation of the *Gem *locus when combined with the inducible *Sox10iCreER* ^*T2*^ line. Whole-mount gut preparations of control (A) and *Sox10CreER(i8.5)|Gem* (B) E12.5 embryos, immunostained for GFP to visualise the distribution of ENCCs within the gut. Red arrows indicate the position of the most caudally located ENCCs in the gut preparations. (C–D), Relative quantitation of *Gem* transcript levels in the FACS-purified ENCCs of *Sox10CreER(i8.5)|Gem* and *Sox10CreER(i10)|Gem* embryos normalised to the levels of b-actin. Unpaired t-test with Welch’s correction, ***P*_value_ < 0.01, ****P*_value_ < 0.001. Scale bar: (A, B) 400 μm. (TIF 1130 kb)
Additional file 6:
**Figure S5.** Ablation of *Gem* from ENCCs does not affect their commitment to the neuronal and glial lineages. (A), Quantification of short-term cultured GFP^+^ ENCCs of control and *Sox10CreER(i10)|Gem* embryos, immunostained for GFP, Ki67, pH3, Sox10, BFABP, S100, Tuj1, HuC/D and processed for EdU labelling. Unpaired t-test, insignificant differences observed. (B–D), Relative quantitation of *Sox10*, *Phox2b*, *Ret*, *HuC* and *BFABP* transcript levels in the ENCCs of *Sox10CreER(i10)|Gem* embryos from the stomach, midgut and caecum-hindgut, respectively. Values were normalised to the levels of GFP. Multiple t-tests without correction, insignificant differences observed. (TIF 1381 kb)
Additional file 7:
**Figure S6.** ENS progenitors proliferate extensively both before and after gut invasion. Cryosections at the level of the foregut and midgut of *Wnt1Cre|EYFP* E9.5 (A) and E11.5 (B) embryos, immunostained for GFP (green) and Ki67 (red). (C), Quantification of NC (GFP^+^) cells positive for Ki67. Unpaired t-test with Welch’s correction, insignificant difference observed. (D), Comparison between the fold decrease in γH2AX^+^ NC cells between *Wnt1Cre|Gem* at E9.5 and *Sox10CreER(i10)|Gem* E11.5 embryos (refer to Figs. 2 and 6) and Ki67^+^ NC cells between *Wnt1Cre|EYFP* at E9.5 and *Wnt1|EYFP* at E11.5. Scale bar: (A, B) 100 μm. (TIF 2506 kb)
Additional file 8:
**Figure S7.** γ-irradiation of E10.0 embryos results in upregulation of γH2AX across the whole tissue. Cryosections at the level of the foregut of *Wnt1Cre|EYFP* control embryos (A, B) or *Wnt1Cre|EYFP* embryos 2 hours after exposure to IR (C, D), immunostained for γH2AX. Stars in A and B indicate the magnified areas (B, D) next to the big panels. Scale bars: (A, C) 100 μm, (B, D) 25 μm. (TIF 1943 kb)
Additional file 9:
**Figure S8.** ENS progenitors established in the gut are resistant to γ-irradiation in the first 2 hours following exposure. Cryosections at the level of the foregut of *Wnt1Cre|EYFP* control E10.5 embryos (A, B) or *Wnt1Cre|EYFP* embryos 2 hours (C, D) or 5 hours (E, F) following exposure to irradiation, immunostained for GFP (green) and processed for TUNEL (red). (G), Quantification of GFP^+^ ENCCs that are undergoing apoptosis (TUNEL^+^). One-way ANOVA followed by Fisher’s LSD multiple comparison test, *****P*_value_ < 0.0001. Scale bar: (A–F) 100 μm. (XLSX 16 kb)

## References

[1] Lee Y (2012). Neurogenesis requires TopBP1 to prevent catastrophic replicative DNA damage in early progenitors. Nat Neurosci.

[2] Liu JC (2013). High mitochondrial priming sensitizes hESCs to DNA-damage-induced apoptosis. Cell Stem Cell.

[3] Milyavsky M (2010). A distinctive DNA damage response in human hematopoietic stem cells reveals an apoptosis-independent role for p53 in self-renewal. Cell Stem Cell.

[4] Hall BK (2008). The neural crest and neural crest cells: discovery and significance for theories of embryonic organization. J Biosci.

[5] Bhatt S, Diaz R, Trainor PA. Signals and switches in Mammalian neural crest cell differentiation. Cold Spring Harb Perspect Biol. 2013;5(2). pii: a008326. 10.1101/cshperspect.a008326 PMC3552505 23378583

[6] Furness JB (2012). The enteric nervous system and neurogastroenterology. Nat Rev Gastroenterol Hepatol.

[7] Laranjeira C, Pachnis V (2009). Enteric nervous system development: recent progress and future challenges. Auton Neurosci.

[8] Barlow AJ (2008). Critical numbers of neural crest cells are required in the pathways from the neural tube to the foregut to ensure complete enteric nervous system formation. Development.

[9] Amiel J (2008). Hirschsprung disease, associated syndromes and genetics: a review. J Med Genet.

[10] Britsch S (2001). The transcription factor Sox10 is a key regulator of peripheral glial development. Genes Dev.

[11] Kapur RP (1999). Early death of neural crest cells is responsible for total enteric aganglionosis in Sox10(Dom)/Sox10(Dom) mouse embryos. Pediatr Dev Pathol.

[12] Teng L (2008). Requirement for Foxd3 in the maintenance of neural crest progenitors. Development.

[13] Durbec PL (1996). Common origin and developmental dependence on c-ret of subsets of enteric and sympathetic neuroblasts. Development.

[14] Pattyn A (1999). The homeobox gene Phox2b is essential for the development of autonomic neural crest derivatives. Nature.

[15] Mundell NA (2012). Enteric nervous system specific deletion of Foxd3 disrupts glial cell differentiation and activates compensatory enteric progenitors. Dev Biol.

[16] Chalazonitis A, Gershon MD, Greene LA (2012). Cell death and the developing enteric nervous system. Neurochem Int.

[17] Kroll KL (2007). Geminin in embryonic development: coordinating transcription and the cell cycle during differentiation. Front Biosci..

[18] Stathopoulou A (2016). Inactivation of Geminin in neural crest cells affects the generation and maintenance of enteric progenitor cells, leading to enteric aganglionosis. Dev Biol.

[19] Karamitros D (2010). Differential geminin requirement for proliferation of thymocytes and mature T cells. J Immunol.

[20] Srinivas S (2001). Cre reporter strains produced by targeted insertion of EYFP and ECFP into the ROSA26 locus. BMC Dev Biol..

[21] Danielian PS (1998). Modification of gene activity in mouse embryos in utero by a tamoxifen-inducible form of Cre recombinase. Curr Biol.

[22] Laranjeira C (2011). Glial cells in the mouse enteric nervous system can undergo neurogenesis in response to injury. J Clin Invest.

[23] Donehower LA (1992). Mice deficient for p53 are developmentally normal but susceptible to spontaneous tumours. Nature.

[24] Scholzen T, Gerdes J (2000). The Ki-67 protein: from the known and the unknown. J Cell Physiol.

[25] Hans F, Dimitrov S (2001). Histone H3 phosphorylation and cell division. Oncogene.

[26] Yu Y (2009). EdU incorporation is an alternative non-radioactive assay to [(3)H]thymidine uptake for in vitro measurement of mice T-cell proliferations. J Immunol Methods.

[27] Van Hooser A (1998). Histone H3 phosphorylation is required for the initiation, but not maintenance, of mammalian chromosome condensation. J Cell Sci.

[28] Eguren M (2013). The APC/C cofactor Cdh1 prevents replicative stress and p53-dependent cell death in neural progenitors. Nat Commun..

[29] Zhou BB, Elledge SJ (2000). The DNA damage response: putting checkpoints in perspective. Nature.

[30] Kerns SL (2012). Geminin is required for zygotic gene expression at the Xenopus mid-blastula transition. PLoS One.

[31] Li A, Blow JJ (2005). Cdt1 downregulation by proteolysis and geminin inhibition prevents DNA re-replication in Xenopus. EMBO J.

[32] Wohlschlegel JA (2000). Inhibition of eukaryotic DNA replication by geminin binding to Cdt1. Science.

[33] Rogakou EP (1998). DNA double-stranded breaks induce histone H2AX phosphorylation on serine 139. J Biol Chem.

[34] Gold R (1994). Differentiation between cellular apoptosis and necrosis by the combined use of in situ tailing and nick translation techniques. Lab Invest.

[35] Mihaylov IS (2002). Control of DNA replication and chromosome ploidy by geminin and cyclin A. Mol Cell Biol.

[36] Zhu W, Chen Y, Dutta A (2004). Rereplication by depletion of geminin is seen regardless of p53 status and activates a G2/M checkpoint. Mol Cell Biol.

[37] Shinnick KM, Eklund EA, McGarry TJ (2010). Geminin deletion from hematopoietic cells causes anemia and thrombocytosis in mice. J Clin Invest.

[38] Spella M (2011). Geminin regulates cortical progenitor proliferation and differentiation. Stem Cells.

[39] Young HM, Bergner AJ, Muller T (2003). Acquisition of neuronal and glial markers by neural crest-derived cells in the mouse intestine. J Comp Neurol.

[40] Barlow A, de Graaff E, Pachnis V (2003). Enteric nervous system progenitors are coordinately controlled by the G protein-coupled receptor EDNRB and the receptor tyrosine kinase RET. Neuron.

[41] Stewart AL, Anderson RB, Young HM (2003). Characterization of lacZ-expressing cells in the gut of embryonic and adult DbetaH-nlacZ mice. J Comp Neurol.

[42] Hayashi S, McMahon AP (2002). Efficient recombination in diverse tissues by a tamoxifen-inducible form of Cre: a tool for temporally regulated gene activation/inactivation in the mouse. Dev Biol.

[43] Nowak E (2006). Radiation-induced H2AX phosphorylation and neural precursor apoptosis in the developing brain of mice. Radiat Res.

[44] Klotz-Noack K (2012). Re-replication induced by geminin depletion occurs from G2 and is enhanced by checkpoint activation. J Cell Sci.

[45] Barry KA (2012). Geminin is required for mitotic proliferation of spermatogonia. Dev Biol.

[46] Symeonidou IE, Taraviras S, Lygerou Z (2012). Control over DNA replication in time and space. FEBS Lett.

[47] Wallace AS (2009). Inhibition of cell death results in hyperganglionosis: implications for enteric nervous system development. Neurogastroenterol Motil.

[48] Uesaka T (2008). Diminished Ret expression compromises neuronal survival in the colon and causes intestinal aganglionosis in mice. J Clin Invest.

[49] Young HM (2001). GDNF is a chemoattractant for enteric neural cells. Dev Biol.

[50] Fonseca-Pereira D (2014). The neurotrophic factor receptor RET drives haematopoietic stem cell survival and function. Nature.

[51] Harfouche G (2010). Fibroblast growth factor type 2 signaling is critical for DNA repair in human keratinocyte stem cells. Stem Cells.

[52] Bhardwaj V (2013). Modulation of c-Met signaling and cellular sensitivity to radiation: potential implications for therapy. Cancer.

[53] Kriegs M (2010). The epidermal growth factor receptor modulates DNA double-strand break repair by regulating non-homologous end-joining. DNA Repair (Amst).

[54] Fernandez RM (2013). Pathways systematically associated to Hirschsprung's disease. Orphanet J Rare Dis..

[55] Laurent A, Blasi F (2015). Differential DNA damage signalling and apoptotic threshold correlate with mouse epiblast-specific hypersensitivity to radiation. Development.

[56] Suberbielle E (2013). Physiologic brain activity causes DNA double-strand breaks in neurons, with exacerbation by amyloid-beta. Nat Neurosci.

[57] Hao MM (2012). Early development of electrical excitability in the mouse enteric nervous system. J Neurosci.

[58] Saffrey MJ (2013). Cellular changes in the enteric nervous system during ageing. Dev Biol.

[59] Jurk D (2012). Postmitotic neurons develop a p21-dependent senescence-like phenotype driven by a DNA damage response. Aging Cell.

