# Supplementary figures and images for: Geminin prevents DNA damage in vagal neural crest cells to ensure normal enteric neurogenesis
Source: BMC Biol. 2016 Oct 24;14:94. doi: 10.1186/s12915-016-0314-x (PMC5075986; doi:10.1186/s12915-016-0314-x)

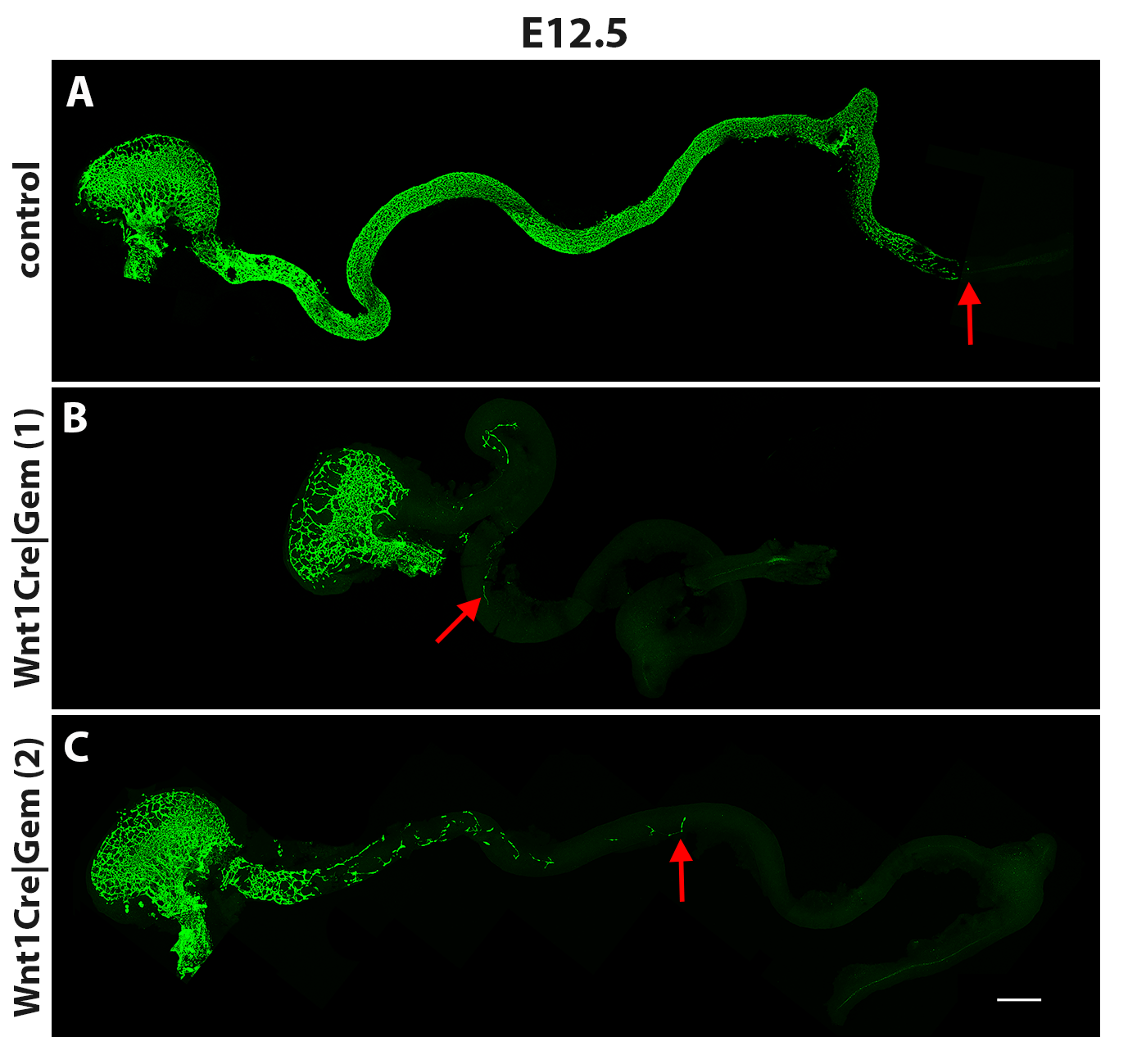

Supplement: Additional file 1: — Raw data. (TIF 482 kb) [file 12915_2016_314_MOESM1_ESM.tif]

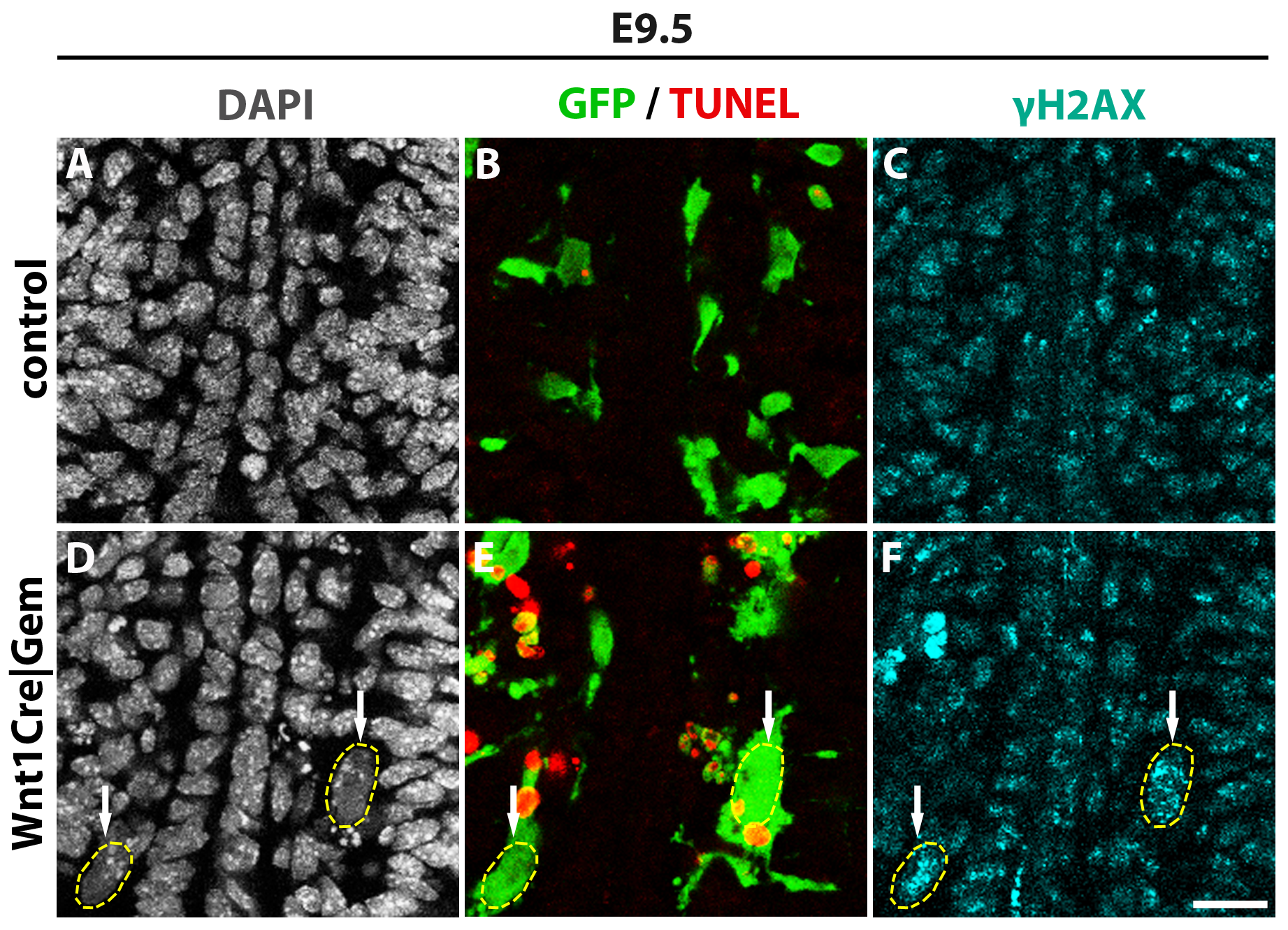

Supplement: Additional file 2: — Figure S1. Ablation of Gem from early pre-ENCCs leads to severe intestinal aganglionosis. Whole-mount gut preparations of control (A) and Wnt1Cre|Gem (B–C) E12.5 embryos immunostained for GFP to visualise migrating ENCCs. Red arrows indicate the position of the most caudally located ENCCs in the gut preparations. Scale bar: (A–C) 400 μm. (TIF 2895 kb) [file 12915_2016_314_MOESM2_ESM.tif]

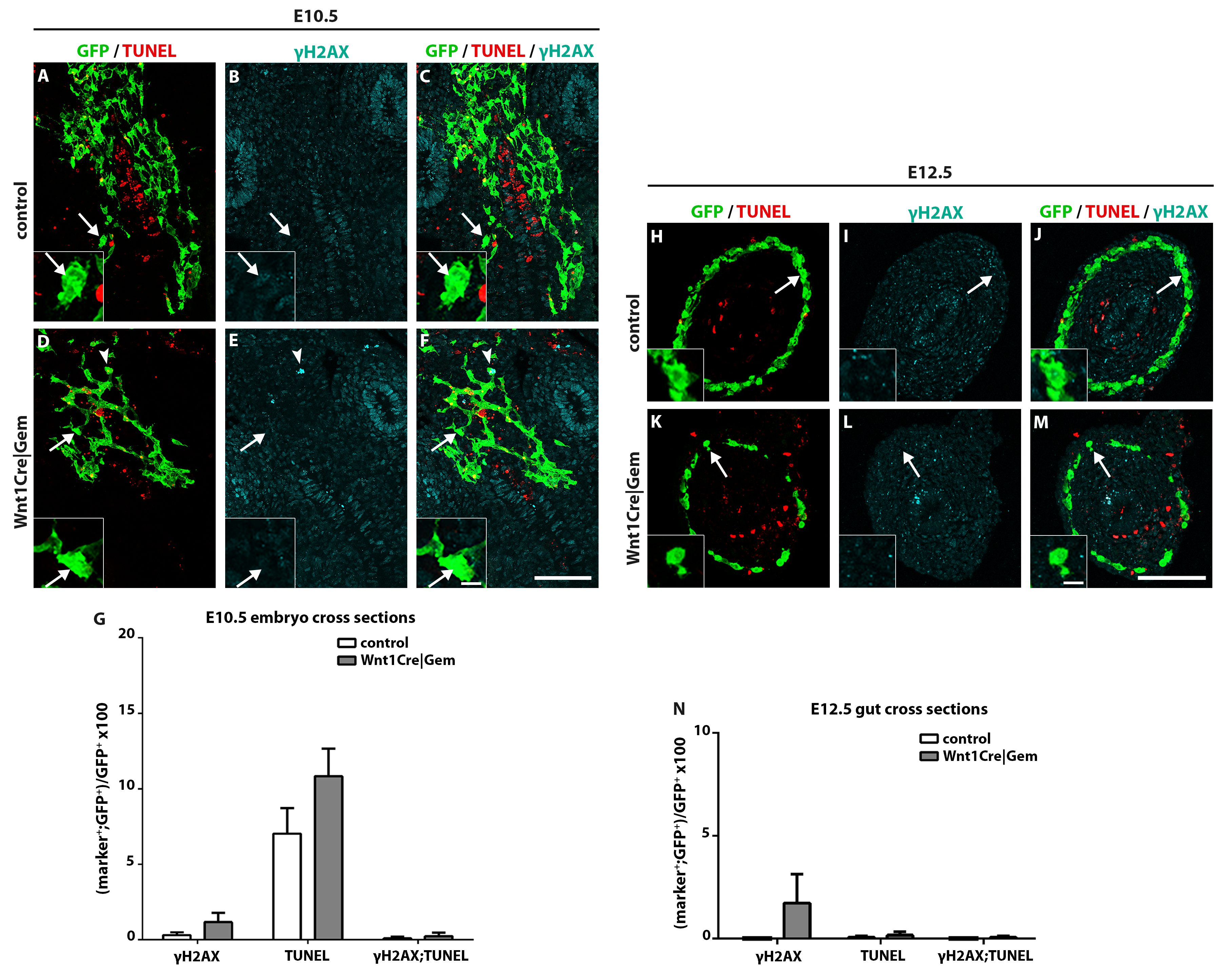

Supplement: Additional file 3: — Figure S2. Ablation of Gem from early pre-ENCCs leads to DNA damage associated with enlargement of the affected nuclei. Cryosections at the level of the foregut of control (A–C) and Wnt1Cre|Gem (D–F) E9.5 embryos, immunostained for GFP (green), γH2AX (cyan), processed for TUNEL (red) and counterstained with DAPI (grey). White arrows and yellow dotted line mark the enlarged nucleus of DNA damaged NC cells that are not undergoing apoptosis. Scale bar: (A–F) 20 μm. (TIF 6057 kb) [file 12915_2016_314_MOESM3_ESM.tif]

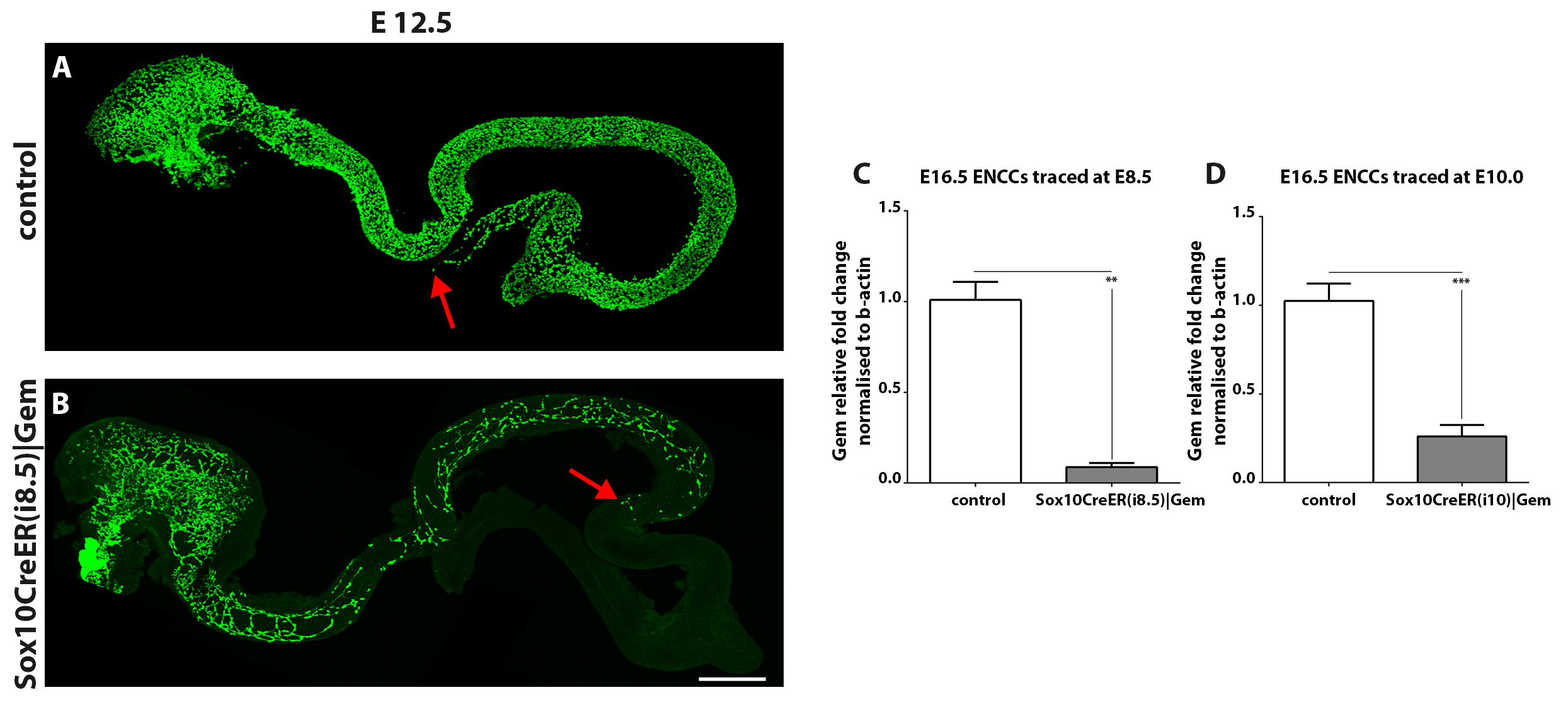

Supplement: Additional file 4: — Figure S3. Ablation of Gem from early pre-ENCCs results in elimination of DNA-damaged and apoptotic cells. Embryo cryosections at the level of the stomach and gut cryosections at the level of the midgut of control (A–C and H–J) and Wnt1Cre|Gem (D–F and K–M) E10.5 and E12.5 embryos, respectively, immunostained for GFP (green), γH2AX (cyan) and processed for TUNEL (red). (G, N) Quantification of NC (GFP+) cells that have DNA damage (γH2AX+), undergo apoptosis (TUNEL+) or both (γH2AX+;TUNEL+). Insets correspond to the areas in the panels indicated with arrows. Multiple t-tests without correction, insignificant differences observed. Scale bars: (A–F, H–M) 100 μm, (insets) 10 μm. (TIF 702 kb) [file 12915_2016_314_MOESM4_ESM.tif]

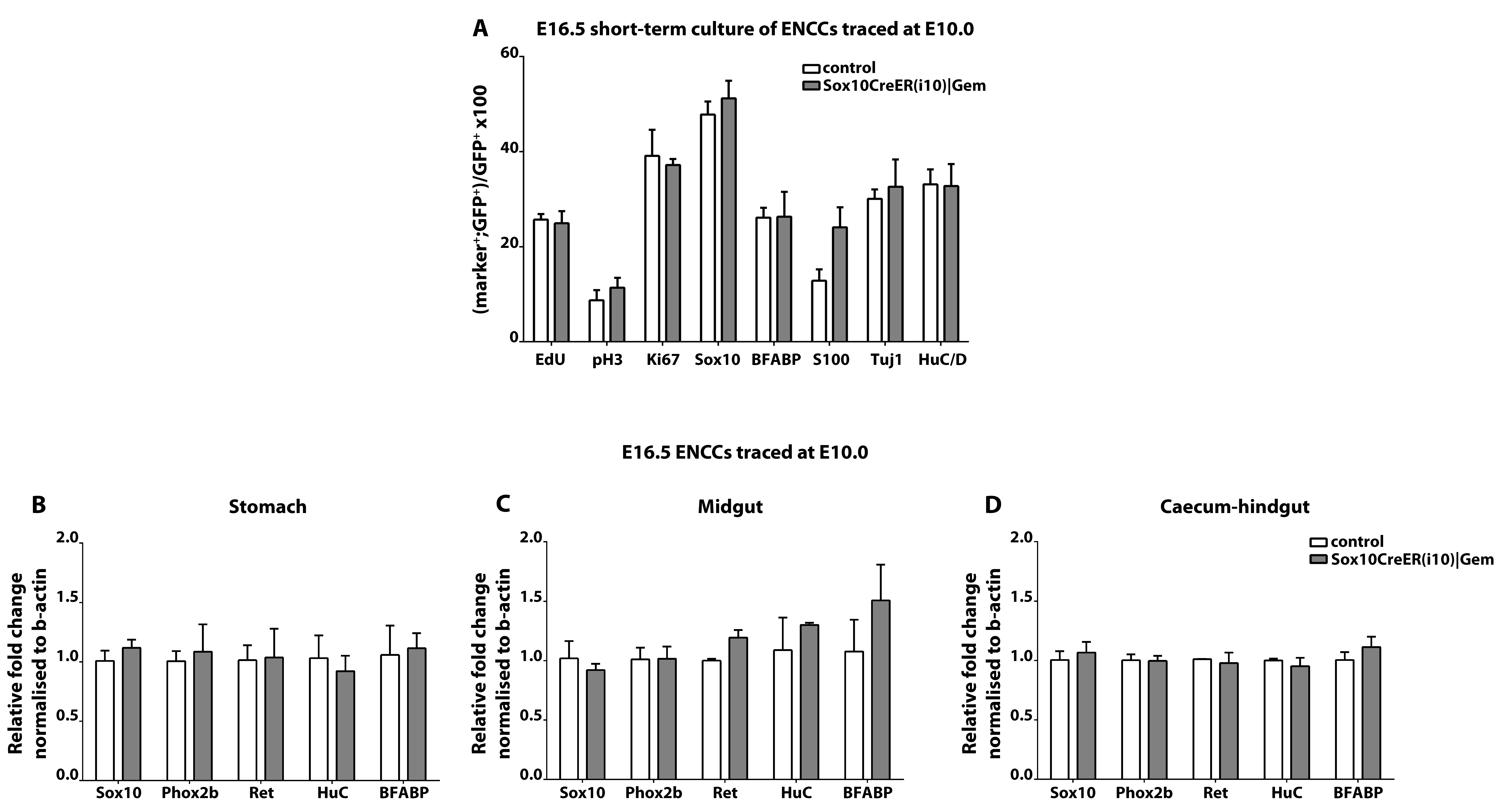

Supplement: Additional file 5: — Figure S4. Efficient ablation of the Gem locus when combined with the inducible Sox10iCreER T2 line. Whole-mount gut preparations of control (A) and Sox10CreER(i8.5)|Gem (B) E12.5 embryos, immunostained for GFP to visualise the distribution of ENCCs within the gut. Red arrows indicate the position of the most caudally located ENCCs in the gut preparations. (C–D), Relative quantitation of Gem transcript levels in the FACS-purified ENCCs of Sox10CreER(i8.5)|Gem and Sox10CreER(i10)|Gem embryos normalised to the levels of b-actin. Unpaired t-test with Welch’s correction, **P value < 0.01, ***P value < 0.001. Scale bar: (A, B) 400 μm. (TIF 1130 kb) [file 12915_2016_314_MOESM5_ESM.tif]

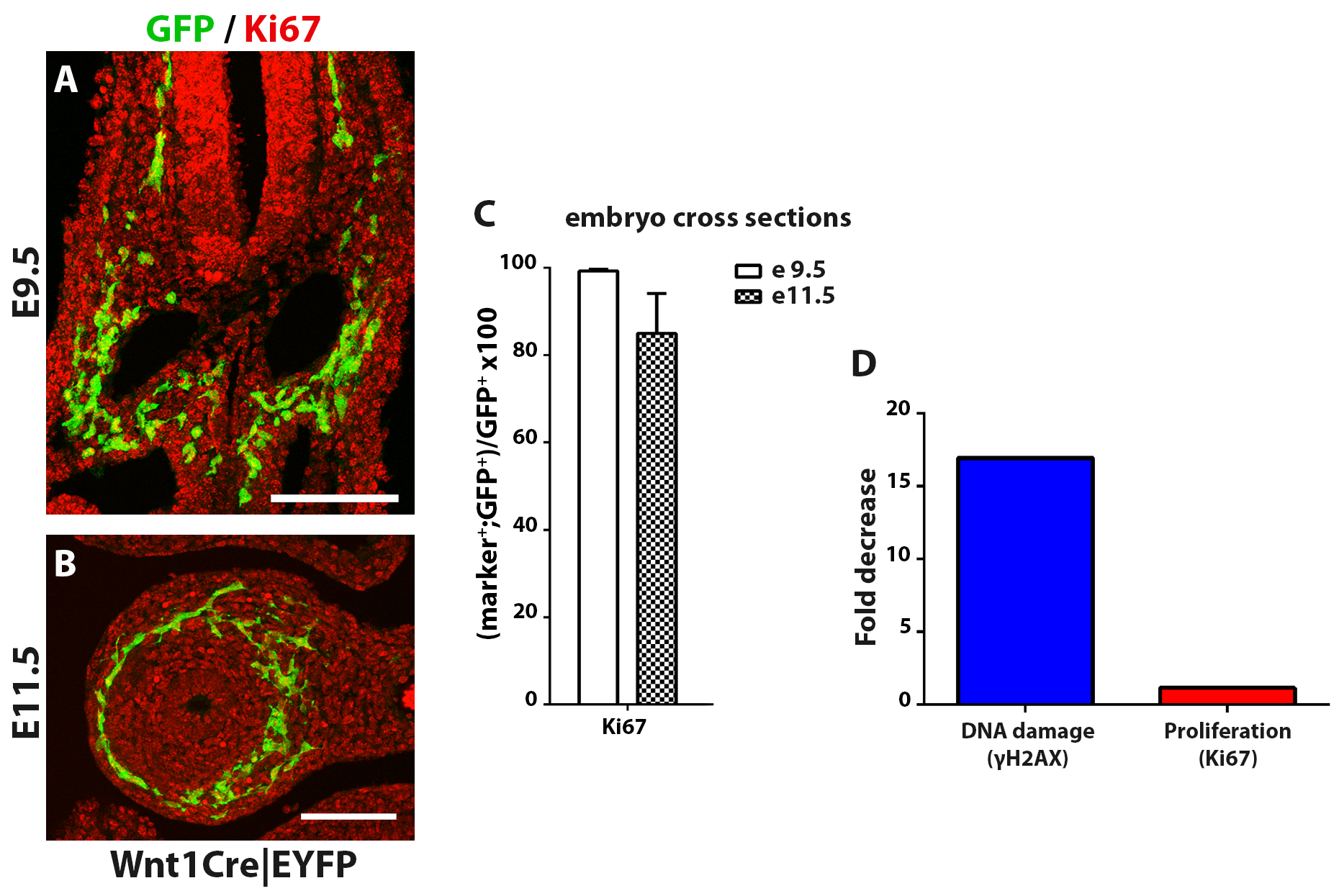

Supplement: Additional file 6: — Figure S5. Ablation of Gem from ENCCs does not affect their commitment to the neuronal and glial lineages. (A), Quantification of short-term cultured GFP+ ENCCs of control and Sox10CreER(i10)|Gem embryos, immunostained for GFP, Ki67, pH3, Sox10, BFABP, S100, Tuj1, HuC/D and processed for EdU labelling. Unpaired t-test, insignificant differences observed. (B–D), Relative quantitation of Sox10, Phox2b, Ret, HuC and BFABP transcript levels in the ENCCs of Sox10CreER(i10)|Gem embryos from the stomach, midgut and caecum-hindgut, respectively. Values were normalised to the levels of GFP. Multiple t-tests without correction, insignificant differences observed. (TIF 1381 kb) [file 12915_2016_314_MOESM6_ESM.tif]

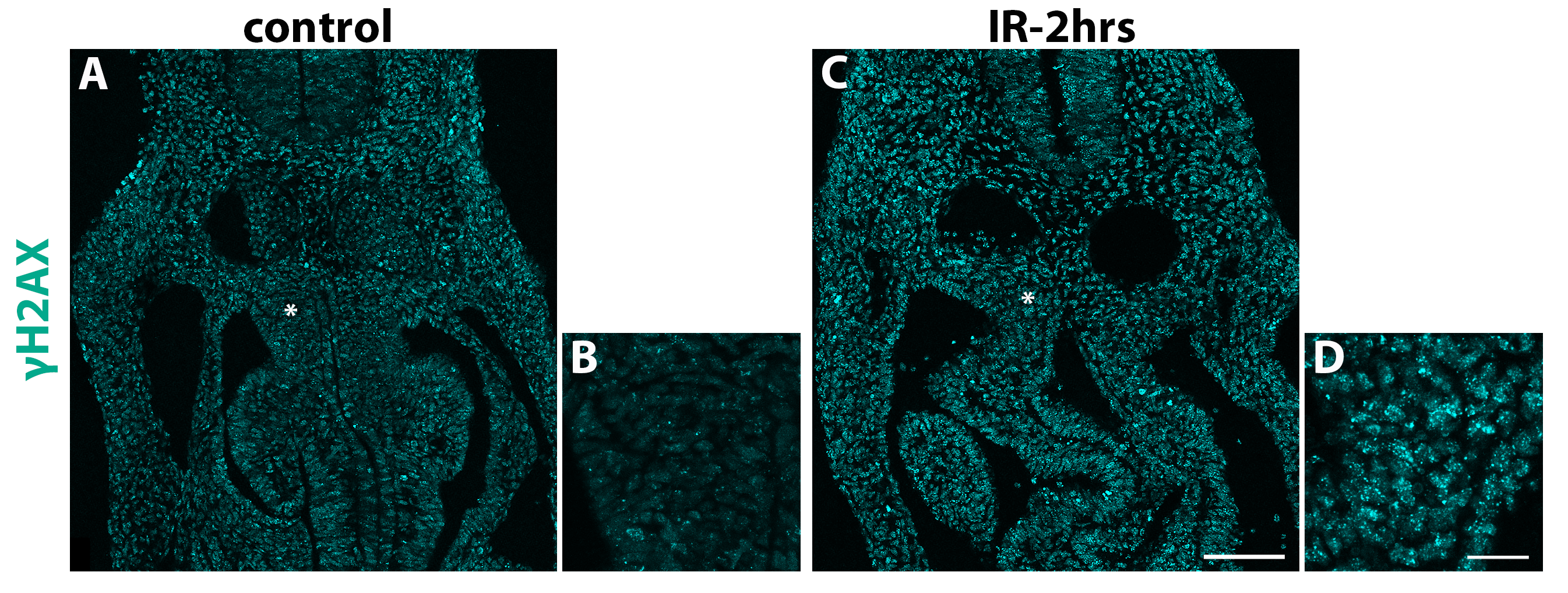

Supplement: Additional file 7: — Figure S6. ENS progenitors proliferate extensively both before and after gut invasion. Cryosections at the level of the foregut and midgut of Wnt1Cre|EYFP E9.5 (A) and E11.5 (B) embryos, immunostained for GFP (green) and Ki67 (red). (C), Quantification of NC (GFP+) cells positive for Ki67. Unpaired t-test with Welch’s correction, insignificant difference observed. (D), Comparison between the fold decrease in γH2AX+ NC cells between Wnt1Cre|Gem at E9.5 and Sox10CreER(i10)|Gem E11.5 embryos (refer to Figs. 2 and 6) and Ki67+ NC cells between Wnt1Cre|EYFP at E9.5 and Wnt1|EYFP at E11.5. Scale bar: (A, B) 100 μm. (TIF 2506 kb) [file 12915_2016_314_MOESM7_ESM.tif]

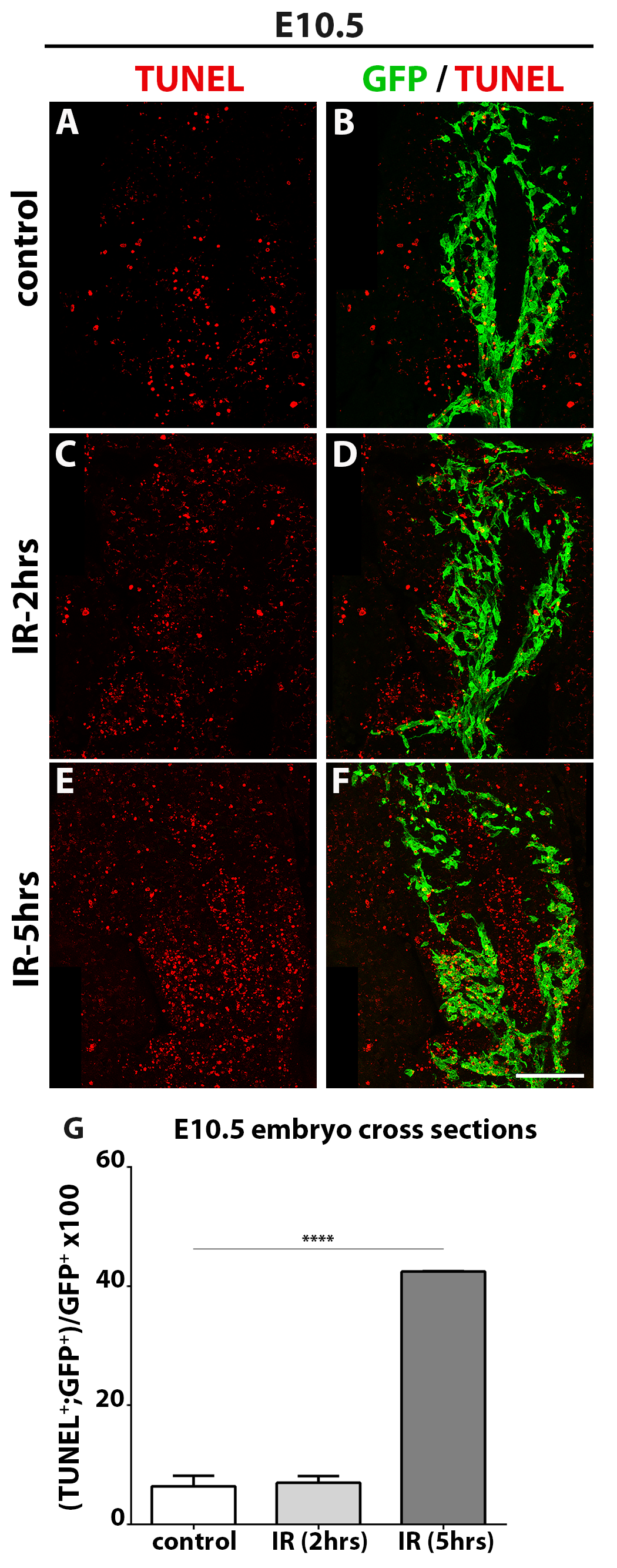

Supplement: Additional file 8: — Figure S7. γ-irradiation of E10.0 embryos results in upregulation of γH2AX across the whole tissue. Cryosections at the level of the foregut of Wnt1Cre|EYFP control embryos (A, B) or Wnt1Cre|EYFP embryos 2 hours after exposure to IR (C, D), immunostained for γH2AX. Stars in A and B indicate the magnified areas (B, D) next to the big panels. Scale bars: (A, C) 100 μm, (B, D) 25 μm. (TIF 1943 kb) [file 12915_2016_314_MOESM8_ESM.tif]
